# Algorithms of causal inference for the analysis of effective connectivity among brain regions

**DOI:** 10.3389/fninf.2014.00064

**Published:** 2014-07-02

**Authors:** Daniel Chicharro, Stefano Panzeri

**Affiliations:** ^1^Neural Computation Laboratory, Center for Neuroscience and Cognitive Systems@UniTn, Istituto Italiano di TecnologiaRovereto, Italy; ^2^Institute of Neuroscience and Psychology, University of GlasgowGlasgow, UK

**Keywords:** causal inference, brain effective connectivity, Pearl causality, Granger causality, Dynamic Causal Models, graphical models, latent processes, spatial aggregation

## Abstract

In recent years, powerful general algorithms of causal inference have been developed. In particular, in the framework of Pearl’s causality, algorithms of inductive causation (IC and IC^*^) provide a procedure to determine which causal connections among nodes in a network can be inferred from empirical observations even in the presence of latent variables, indicating the limits of what can be learned without active manipulation of the system. These algorithms can in principle become important complements to established techniques such as Granger causality and Dynamic Causal Modeling (DCM) to analyze causal influences (effective connectivity) among brain regions. However, their application to dynamic processes has not been yet examined. Here we study how to apply these algorithms to time-varying signals such as electrophysiological or neuroimaging signals. We propose a new algorithm which combines the basic principles of the previous algorithms with Granger causality to obtain a representation of the causal relations suited to dynamic processes. Furthermore, we use graphical criteria to predict dynamic statistical dependencies between the signals from the causal structure. We show how some problems for causal inference from neural signals (e.g., measurement noise, hemodynamic responses, and time aggregation) can be understood in a general graphical approach. Focusing on the effect of spatial aggregation, we show that when causal inference is performed at a coarser scale than the one at which the neural sources interact, results strongly depend on the degree of integration of the neural sources aggregated in the signals, and thus characterize more the intra-areal properties than the interactions among regions. We finally discuss how the explicit consideration of latent processes contributes to understand Granger causality and DCM as well as to distinguish functional and effective connectivity.

## Introduction

The need to understand how the interactions and coordination among brain regions contribute to brain functions has led to an ever increasing attention to the investigation of brain connectivity (Bullmore and Sporns, 2009; Friston, 2011). In addition to anatomical connectivity, two other types of connectivity that regard how the dynamic activity of different brain regions is interrelated have been proposed. *Functional connectivity* refers to the statistical dependence between the activity of the regions, while *effective connectivity* refers, in a broad sense, to the causal influence one neural system exerts over another (Friston, 2011).

Attempts to go beyond the study of dynamic correlations to investigate the causal interactions among brain regions have made use of different approaches to study causality developed outside neuroscience (Granger, 1963, 1980). Granger causality was proposed in econometrics to infer causality from time-series and has been widely applied in neuroscience as a model-free approach to study causal interactions among brain regions (see Bressler and Seth, 2011, for an overview). It has been applied to different types of neural data, from intracranial electrophysiological recordings (e.g., Bernasconi and König, 1999; Besserve et al., 2010), Magnetoencephalography recordings (e.g., Vicente et al., 2011), to functional magnetic resonance imaging (fMRI) measures (e.g., Roebroeck et al., 2005; Mäki-Marttunen et al., 2013; Wu et al., 2013). New approaches have been also developed within neuroscience, such as Dynamic Causal Modeling (DCM) (Friston et al., 2003) which explicitly models the biophysical interactions between different neural populations as well as the nature of the recorded neural signals (Friston et al., 2013).

Separately, in the field of artificial intelligence, another approach to causal analysis has been developed by Pearl and coworkers. Pearl’s approach combines causal models and causal graphs (Spirtes et al., 2000; Pearl, 2009). The fundamental difference with the approaches currently used to study the brain’s effective connectivity (Granger causality and DCM) is that the understanding of causation in Pearl’s framework ultimately relies on the notion of an external intervention that actively perturbs the system. This notion of intervention provides a rigorous definition of the concept of causal influence but at the same time illustrates the limitations of causal analysis from observational studies.

The analysis of the causal influence one neural system exerts over another (i.e., effective connectivity) requires considering causation at different levels (Chicharro and Ledberg, 2012a), in particular distinguishing between causal inference and quantification or modeling of causal effects (Pearl, 2009). At the most basic level, *causal inference* deals with assessing which causal connections exist and which do not exist, independently of their magnitude or the mechanisms that generate them. At a higher level, the quantification of the magnitude implies selecting a measure of the strength of the causal effect, and the characterization of the mechanisms implies implementing a plausible model of how the dynamics of the system are generated. Recently, it has been pointed out that the existence of causal connections should be distinguished from the existence of causal effects, and in particular that only in some cases it is meaningful to understand the interactions between subsystems in terms of the causal effect one exerts over another (Chicharro and Ledberg, 2012a). Furthermore, the possibility and the limitations to quantify causal influences with Granger causality has been examined (Lizier and Prokopenko, 2010; Chicharro and Ledberg, 2012b; Chicharro, 2014b).

In this work we focus on the basic level of causal analysis constituted by causal inference. In particular, we investigate how some general algorithms of causal inference (IC and IC^*^ algorithms) developed in the Pearl’s framework (Verma and Pearl, 1990; Pearl, 2009) can be applied to infer causality between dynamic processes and thus used for the analysis of effective connectivity. This algorithmic approach relies on the evaluation of the statistical dependencies present in the data, similarly to the non-parametric formulation of Granger causality. Its particularity is that it explicitly considers the impact of latent (unobserved) processes as well as the existence of different causal structures which are equivalent in terms of the statistical dependencies they produce. Accordingly, it provides a principled procedure to evaluate the discrimination power of the data with respect to the possible causal structures underlying the generation of these data.

Although these causal algorithms do not assume any constraint on the nature of the variables to which they are applied, their application to dynamic processes has yet to be investigated. The main goal of this paper is to study the extension of Pearls causal approach to dynamic processes and to evaluate conceptually how it can contribute to the analysis of effective neural connectivity. To guide the reader, we provide below an overview of the structure of this article.

## Overview of the structure of the article

We start by reviewing the approach to causal inference of Pearl (2009) and Granger (1963, 1980) and we then focus on the analysis of temporal dynamics. In the first part of our Results we investigate the application to dynamic processes of the algorithms of causal inference proposed by Pearl. We then recast their basic principles combining them with Granger causality into a new algorithm which, as the IC^*^ algorithm, explicitly deals with latent processes but furthermore provides a more suited output representation of the causal relations among the dynamic processes.

In the second part of our Results, we shift the focus from the inference of an unknown causal structure to studying how statistical dependencies can be predicted from the causal structure. In particular, for a known (or hypothesized) causal structure underlying the generation of the recorded signals, we use graphical criteria to identify the statistical dependencies between the signals. We specifically consider causal structures compatible with the state-space models which have recently been recognized as an integrative framework in which refinements of Granger causality and DCM converge (Valdes-Sosa et al., 2011). This leads us to reformulate in a general unifying graphical approach different effects relevant for the analysis of effective connectivity, such as those of measurement noise (Nalatore et al., 2007), of hemodynamic responses (e.g., Seth et al., 2013), and of time aggregation (e.g., Smirnov, 2013). We especially focus on the effect of spatial aggregation caused by the superposition in the recorded signals of the massed activity of the underlying sources of neural activity interacting at a finer scale.

Finally, in Discussion we discuss the necessity to understand how causal interactions propagate from the microscopic to the macroscopic scale. We indicate that, although the algorithms here discussed constitute a non-parametric approach to causal inference, our results are also relevant for modeling approaches such as DCM and help to better understand how difficult it is in practice to distinguish functional and effective connectivity.

## Review of relevant concepts of causal models

In this section, we lay the basis for the novel results by reviewing the approach to causal inference of Pearl (2009) and Granger (1963, 1980).

### Models of causality

We begin reviewing the models of causality described by Pearl (2009) and relating them to DCM (Friston et al., 2003). For simplicity, we restrict ourselves to the standard Pearl models which are the basis of the IC and IC^*^ algorithm, without reviewing extensions of these models such as settable systems (White and Chalak, 2009), which are suitable for a broader set of systems involving, e.g., optimization and learning problems.

A *Causal Model M* is composed by a set of *n* stochastic variables *V*_*k*_, with *k* ∈ {1, …, *n*} which are endogenous to the model, and a set of *n*′ stochastic variables *U*′_*k*_, with *k*′ ∈ {1, …, *n*′}, which are exogenous to the model. Endogenous variables are those explicitly observed and modeled. For example, when studying the brain’s effective connectivity, these variables may be the neural activity of a set of *n* different regions. The exogenous variables correspond to sources of variability not explicitly considered in the model, which can for example correspond to sources of neuromodulation, uncontrolled variables related to changes in the cognitive state (Masquelier, 2013), or activity of brain areas not recorded. Accordingly, for each variable *V*_*k*_ the model contains a function *f_k_* such that

(1) $$V_{k}=f_{k}(pa(V_{k}),U_{k},\theta_{k})$$

That is, the value of *V_k_* is assigned by a function *f_k_* determined by a set **θ**_*k*_ of constant parameters and taking as arguments a subset of the endogenous variables which is called the *parents* of *V_k_* (*pa*(*V_k_*)), as well as a subset of the exogenous variables ***U***_*k*_. In general, in Pearl’s formulation the exogenous variables are considered as noise terms which do not introduce dependencies between the endogenous variables, so that a single variable ***U***_*k*_ can be related to each *V_k_*. Causality from *V_j_* to *V*_*j*′_ is well-defined inside the model: *V_j_* is directly causal to *V*_*j*′_ if it appears as an argument of the function *f*_*j*′_, that is, if *V_j_* is a parent of *V*_*j*′_ (*V_j_* ∈ *pa*(*V*_*j*′_)). However, whether the inside-model causal relation correctly captures some real physical causality depends on the goodness of the model. To complete the model the probability distribution *p*({*U*}) of the exogenous variables is required, so that the joint distribution of the endogenous variables *p*({*V*}) is generated using the functions. Accordingly, *p*({*V*}) can be decomposed in a Markov factorization that reflects the constraints in terms of conditional independence that result from the functional model:

(2) $$p(V_{1},\ldots ,V_{n})=\prod_{k=1}^{n}p(V_{k}|pa(V_{k})).$$

Each causal model *M* has an associated graphical representation called *causal structure* G(*M*). A causal structure is a directed acyclic graph (DAG) in which each endogenous variable *V_k_* corresponds to a node and an arrow pointing to *V_k_* from each of its parents is added. A *path* between nodes *V_j_* and *V*_*j*′_ is a sequence of arrows linking *V_j_* and *V*_*j*′_. It is not required to follow the direction of the arrows, and a path that respects their direction is called a *directed path*. A causal structure reflects the parental structure in the functional model, and thus indicates some constraints to the set **Θ** = {**θ**_1_, …, **θ**_*n*_} of constant parameters used to construct the functions. The factorization of Equation (2) is reflected in *V_k_* being conditionally independent from any other of its ancestors once conditioned on *pa*(*V_k_*), where the ancestors of *V_k_*—i.e., *an* (*V_k_*)—are defined in the graph as those nodes that can be attained by following backwards any directed path that arrives to *V_k_*.

In the formulation of Pearl no constraints concern the nature of the variables in the causal model. However, in the presentation of Pearl’s framework (Pearl, 2009) dynamic variables are seldom used. This fact, together with the fact that the causal graphs associated with the causal models are acyclic, has sometimes lead to erroneously think that the Pearl’s formulation is not compatible with processes that involve feedback connections, since they lead to cyclic structures in the graph (see Valdes-Sosa et al., 2011, for discussion). However, cycles only appear when not considering the dynamic nature of the causal model underlying the graphical representation. For dynamic variables, the functional model consists of a set of differential equations, DCM state equations being a well-known example (Valdes-Sosa et al., 2011). In particular, in a discretized form, the state equations are expressed as

(3) $$V_{k,i+1}=f_{k}(pa(V_{k,i+1}),U_{k,i};\theta_{k});$$

where *V*_*k,i*+1_ is the variable associated with the time sampling *i*+1 of process *k*. In general, the parents of *V*_*k,i*+1_ include *V*_*k,i*_ and can comprise several sampling times from other processes, depending on the delay in the interactions. Depending on the type of DCM models used, deterministic or stochastic, the variables {*U*} can comprise exogenous drivers or noise processes. It is thus clear that the models of causality described by Pearl are general and comprise models of the form used in DCM.

### Statistical independencies determined by causal interactions

As mentioned above, a causal structure is a graph that represents the structure of the parents in a causal model. Pearl (1986) provided a graphical criterion for DAGs called *d-separation*—where *d* stands for directional—to check the independencies present in any model compatible with a causal structure. Its definition relies on the notion of *collider* on a path, a node on a path for which, when going along the path, two arrows point toward the node (→ *V* ←). The criterion of d-separation states:

#### D-separation

Two nodes *V_j_*, *V*_*j*′_ are d-separated by a set of nodes *C* if and only if for every path between *V_j_*, *V*_*j*′_ one of the following conditions is fulfilled:

1. The path contains a non-collider *V_k_* (→ *V_k_* → or ← *V_k_*→) which belongs to *C*.
2. The path contains a collider *V_k_* (→ *V_k_* ←) which does not belong to *C* and *V_k_* is not an ancestor of any node in *C*.

For a causal model compatible with a causal structure the d-separation of *V_j_* and *V*_*j*′_ by *C* is a sufficient condition for *V_j_* and *V*_*j*′_ being conditional independent given *C*, that is

(4) $$V_{j}{⊥}_{\text{G}}V_{j^{\prime }}|C\Rightarrow V_{j}{⊥}_{\text{M}}V_{j^{\prime }}|C$$

where ⊥_G_ indicates d-separation in the causal structure *G* and ⊥_M_ independence in the joint probability distribution of the variables generated by the causal model *M*. This sufficient condition can be converted into an if and only if condition if further assuming *stability* (Pearl, 2009)—or equivalently *faithfulness* (Spirtes et al., 2000)—, which states that conditional independence between the variables does not result from a particular tuning of the parameters Θ, which would disappear if those were infinitesimally modified.

**Figure 1:**
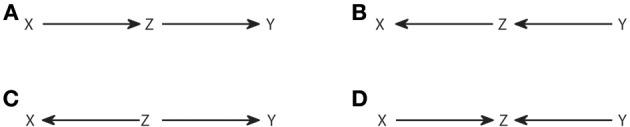
**Observationally equivalent causal structures**. The causal structures **(A–C)** are observationally equivalent, while the one in **(D)** is distinguishable from them.

Considering the correspondence between d-separation and conditional independence, an important question is the degree to which the underlying causal structure can be inferred from the set of conditional independencies present in an observed joint distribution. The answer is that there are classes of causal structures which are observationally equivalent, that is, they produce exactly the same set of conditional independencies observable from the joint distribution. Consider, for example, the four causal structures of Figure 1. Each causal structure is characterized by a list of all the conditional independencies compatible with it. Applying d-separation it can be checked that for Figures 1A–C we have that *X* and *Y* are d-separated by Z (*X*⊥*Y*|*Z*), while in Figure 1D *X* and *Y* are d-separated by the empty set (*X* ⊥ *Y*). Therefore, we can discriminate Figures 1A–C from Figure 1D, but not among Figures 1A–C. Statistical dependencies, the only type of available information when recording the variables, only retain limited information about how the variables have been generated.

Verma and Pearl (1990) provided the conditions for two DAGs to be *observationally equivalent*. Two DAGs are observationally equivalent if and only if they have the same skeleton and the same v-structures, where the skeleton refers to the links without considering the direction of the arrows, and a v-structure refers to three nodes such that two arrows point head to head to the central node, while the other two nodes are *non-adjacent*, i.e., not directly linked (as in Figure 1D). It is clear from this criterion that the structures in Figures 1A–C are equivalent and the one in Figure 1D is not.

### Causal inference

#### Causal inference without latent variables, the IC algorithm

Given the existence of observationally equivalent classes of DAGs, it is clear that there is an intrinsic fundamental limitation to the inference of a causal structure from recorded data. This is so even assuming that there are no latent variables. Here we review the IC algorithm (Verma and Pearl, 1990; Pearl, 2009), which provides a way to identify with which equivalence class a joint distribution is compatible, given the conditional independencies it contains. The input to the algorithm is the joint distribution *p*({*V*}) on the set {*V*} of variables, and the output is a graphical pattern that reflects all and no more conditional independencies than the ones in *p*({*V*}). These independencies can be read from the pattern applying d-separation. The algorithm is as following:

### IC algorithm (inductive causation)

1. For each pair of variables *a* and *b* in {*V*} search for a set *S*_*ab*_ such that conditional independence between *a* and *b* given *S_ab_* (*a* ⊥ *b*|*S_ab_*) holds in *p*({*V*}). Construct an undirected graph linking the nodes *a* and *b* if and only if *S_ab_* is not found.
2. For each pair of non-adjacent nodes *a* and *b* with a common adjacent node *c* check if c belongs to *S_ab_*
  If it does, then continue.
  If it does not, then add arrowheads pointing at *c* to the edges (i.e., *a* → *c* ← *b*).
3. In the partially oriented graph that results, orient as many edges as possible subject to two conditions: (i) Any alternative orientation would yield a new v-structure. (ii) Any alternative orientation would yield a directed cycle.

**Figure 2:**
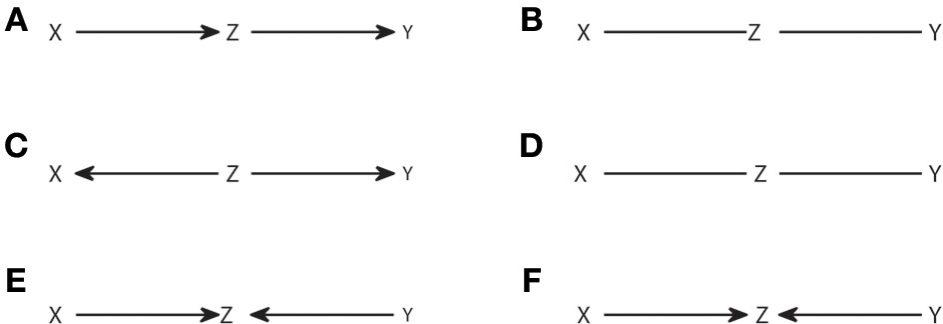
**Causal structures (A,C,E) and their corresponding patterns obtained with the IC algorithm (B,D,F)**.

The algorithm is a straightforward application of the definition of observational equivalence. Step 1 recovers the skeleton of the graph, linking those nodes that are dependent in any context. Step 2 identifies the v-structures and Step 3 prevents creating new ones or cycles. A more procedural formulation of Step 3 was proposed in Verma and Pearl (1992). As an example, in Figure 2 we show the output from the IC algorithm that would result from joint distributions compatible with causal structures of Figure 1. Note that throughout this work, unless otherwise stated, conditional independencies are not evaluated by estimating the probability distributions, but graphically identified using Equation (4). The causal structures of Figures 2A,C result in the same pattern (Figures 2B,D, respectively), which differ from the one that results from Figure 2E (Figure 2F).

The output pattern is not in general a DAG because not all links are arrows. It is a partial DAG which constitutes a graphical representation of the conditional independencies. D-separation is applicable, but now it has to be considered that non-colliders comprise edges without arrows, while the definition of collider remains the same. Note that, to build any causal structure that is an element of the class represented by a pattern, one has to continue adding arrows to the pattern subject to not creating v-structures or cycles. For example, the pattern of Figure 2B can be completed to lead to any causal structure of Figures 1A–C, but one cannot add head to head arrows, because this would give a non-compatible causal structure which corresponds to the pattern of Figure 2F.

### Causal inference with latent variables: the IC^*^ algorithm

So far we have addressed the case in which the joint distribution *p*({*V*}) includes all the variables of the model. Now we consider that only a subset {*V_O_*} is observed. We have seen that while a causal structure corresponds to a unique pattern which represents the equivalence class, a pattern can represent many causal structures. The size of the equivalence class generally increases with the number of nodes. This means that when latent variables are not excluded, if no constraints are imposed to the structure of the latent variables, the size of the class grows infinitely. For example, if the latent variables are interlinked, the unobserved part of the causal structure may contain many conditional independencies that we cannot test. To handle this, Verma (1993) introduced the notion of a *projection* and proved that any causal structure with a subset {*V_O_*} of observable nodes has a dependency-equivalent projection, that is, another causal structure compatible with the same set of conditional independencies involving the observed variables, but for which all unobserved nodes are not linked between them and are parents of exactly two observable nodes. Accordingly, the objective of causal inference with the IC^*^ algorithm is to identify with which dependency-equivalent class of projections a joint distribution *p*({*V_O_*}) is compatible. In the next section we will discuss how relevant it is for the application to dynamic processes the restriction of inference to projections instead of more general causal structures.

The input to the IC^*^ algorithm (Verma, 1993; Pearl, 2009) is *p*({*V_O_*}). The output is an *embedded pattern*, a hybrid acyclic graph that represents all and no more conditional independencies than the ones contained in *p*({*V_O_*}). While the patterns that result from the IC algorithm are partial DAGs which only contain arrows that indicate a causal connection, or undirected edges to be completed, the embedded patterns obtained with the IC^*^ algorithm are hybrid acyclic graphs because they can contain more types of links: genuine causal connections are indicated by solid arrows (*a* → *b*). These are the only causal connections that can be inferred with certainty from the independencies observed. *Potential causes* are indicated by dashed arrows (*a* ⇢ *b*), and refer to a possible causal connection (*a* → *b*), or to a possible latent common driver (*a* ← α → *b*), where greek letters are used for latent nodes. Furthermore, bidirectional arrows indicate certainty about the existence of a common driver. Undirected edges indicate a link yet to be completed. Therefore, there is a hierarchy of inclusion of the links, going from completely undefined, to completely defined identification of the source of the dependence: Undirected edges subsume potential causes, which subsume genuine causes and common drivers.

Analogously to the patterns of the IC algorithm, the embedded patterns are just a graphical representation of the dependency class. Their main property is that using d-separation one can read from the embedded pattern all and no more than the conditional independencies compatible with the class. In the case of the embedded patterns, d-separation has to be applied extending the definition of collider to any head to head arrows of any of the type present in the hybrid acyclic graphs.

### IC^*^ algorithm (inductive causation with latent variables)

1. For each pair of variables *a* and *b* in {*V_O_*} search for a set *S_ab_* such that conditional independence between *a* and *b* given *S_ab_* (*a* ⊥ *b*| *S_ab_*) holds in *p*({*V_O_*}). Construct an undirected graph linking the nodes *a* and *b* if and only if *S_ab_* is not found.
2. For each pair of non-adjacent nodes *a* and *b* with a common adjacent node *c* check if *c* belongs to *S_ab_*
  If it does, then continue.
  If it does not, then substitute the undirected edges by dashed arrows pointing at *c*.
3. Recursively apply the following rules:

- 3R_1_: if *a* and *b* are non-adjacent, they have a common adjacent node *c*, if the link between *a* and *c* has an arrowhead into *c* and the link between *b* and *c* has no arrowhead into *c*, then substitute the link between *c* and *b* (either an undirected edge or a dashed arrow) by a solid arrow from *c* to *b*, indicating a genuine causal connection (*c* → *b*).
- 3R_2_: if there is a directed path from *a* to *b* and another path between them with a link that renders this path compatible with a directed path in the opposite direction, substitute the type of link by the one immediately below in the hierarchy that excludes the existence of a cycle.

Steps 1 and 2 of the algorithm are analogous to the steps of the IC algorithm, except that now in Step 2 dashed arrows are introduced indicating potential causes. The application of step 3 is analogous to the completion in Step 3 of the IC algorithm, but adapted to consider all the types of links that are now possible. In 3R_1_ a causal connection (*c* → *b*) is identified because either a causal connection on the opposite direction or a common driver would create a new v-structure. In 3R_2_ cycles are avoided.

**Figure 3:**
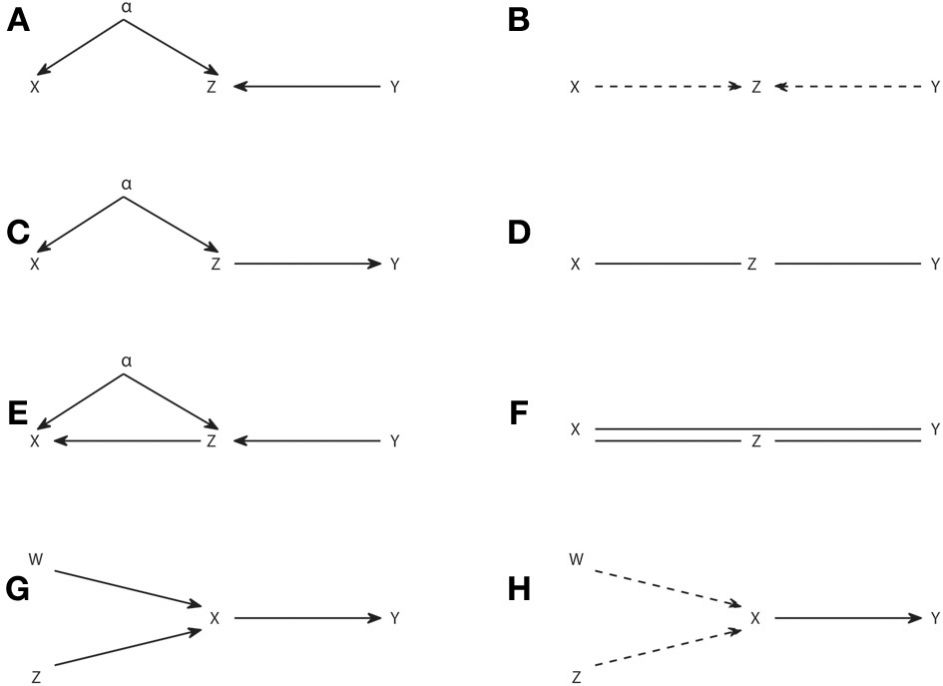
Causal structures containing latent variables (A,C,E,G) and their corresponding embedded patterns obtained with the IC^*^ algorithm (B,D,F,H).

As an example of the application of the IC^*^ algorithm in Figure 3 we show several causal structures and their corresponding embedded patterns. The causal structure of Figure 3A results in an embedded pattern with two potential causes pointing to Z (Figure 3B), while the one of Figure 3C results in an embedded pattern with undirected edges (Figure 3D). The embedded pattern of Figure 3B can be seen as a generalization, when latent variables are considered, of the pattern of Figure 2F. Similarly, the pattern of Figure 3D is a generalization of Figures 2B,D. In the case of these embedded patterns a particular causal structure from the dependency class can be obtained by selecting one of the connections compatible with each type of link, e.g., a direct arrow or to add a node that is a common driver for the case of dashed arrows indicating a potential cause. Furthermore, like for the completion of patterns obtained from the IC algorithm, no new v-structures or cycles can be created, e.g., in Figure 3D the undirected edges cannot be both substituted by head to head arrows.

However, in general for the embedded patterns, not all the elements of the dependency class can be retrieved by completing the links, even if one restricts itself to projections. For example, consider the causal structure of Figure 3E and its corresponding embedded pattern in Figure 3F. In this case the embedded pattern does not share the skeleton with the causal structure, since a link *X*–*Y* is present indicating that *X* and *Y* are adjacent. This makes the mapping of the embedded pattern to the underlying causal structure less intuitive and further highlights that the patterns and embedded patterns are just graphical representations of a given observational and dependency class, respectively.

As a last example in Figures 3G,H we show a causal structure and its corresponding embedded pattern where a genuine causal structure is inferred by applying the rule 3R_1_. A genuine cause from *X* to *Y* (*X* → *Y*) is the only possibility since a genuine cause from *Y* to *X* (*X* ← *Y*), as well as a common driver (*X* ← α → *Y*) would both create a new v-structure centered at *X*. Therefore, rule 3R_1_ reflects that even if allowing for the existence of latent variables, it is sometimes possible to infer a genuine causation just from observations, without having to manipulate the system. As described in rule 3R_1_, inferring genuine causation from a variable *X* to a variable *Y* always involves a third variable and requires checking at least two conditional independencies. See the Supplementary Material for details of a sufficient condition of genuine causation (Verma, 1993; Pearl, 2009) and how it is formulated in terms of Granger causality when examining dynamic processes.

### The criterion of Granger causality for causal inference

So far we have reviewed the approach of Pearl based on models of causality and graphical causal structures. The algorithms of causal inference proposed in this framework are generic and not conceived for a specific type of variables. Conversely, Granger (1963, 1980) proposed a criterion to infer causality specifically between dynamic processes. The criterion to infer causality from process *X* to process *Y* is based on the extra knowledge obtained about the future of *Y* given the past of *X*, in a given context *Z*. In its linear implementation, this criterion results in a comparison of prediction errors, however, as already pointed out by Granger (1980), a strong formulation of the criterion is expressed as a condition of independence

(5) $$p(Y_{i+1}|{\left\{V\right\}}^{i})=p(Y_{i+1}|{\left\{V\right\}}^{i}\backslash X^{i}),$$

where the superindex *i* refers to the whole past of a process up to and including sample *i*, {*V*} refers to the whole system {*X*, *Y*, *Z*}, and {*V*^i^}\*X^i^* refers to the past of the whole system excluding the past of *X*. That is, *X* is Granger non-causal to *Y* given *Z* if the equality above holds. Granger (1980) indicated that Granger causality is context dependent, i.e., adding or removing other processes from the context *Z* affects the test for causality. In particular, genuine causality could only be checked if *Z* was including all the processes that have a causal link to *X* and *Y*, otherwise a hidden common driver or an intermediate process may be responsible for the dependence. Latent variables commonly result in the existence of instantaneous correlations, which are for example reflected in a non-zero cross-correlation of the innovations when multiple regression is used to analyze linear Granger causality. In its strong formulation (Granger, 1980) the existence of instantaneous dependence is tested with the criterion of conditional independence

(6) $$p(X_{i+1},Y_{i+1}|{\left\{V\right\}}^{i})=p(X_{i+1}|{\left\{V\right\}}^{i})p(Y_{i+1}|{\left\{V\right\}}^{i}),$$

called by Granger *instantaneous causality* between *X* and *Y*. Both criteria of Granger causality and instantaneous causality can be generally tested using the conditional Kullback-Leibler divergence (Cover and Thomas, 2006)

(7) $$KL(p(Y|X);q(Y|X))=\sum_{x,y}p(x,y)\log\frac{p(y|x)}{q(y|x)}.$$

The KL-divergence is non-negative and only zero if the distributions *p* and *q* are equal. Accordingly, plugging into Equation (7) the probability distributions of the criterion of Granger causality of Equation (5) we get (Marko, 1973).

(8) $$\begin{array}{l} T_{X\to Y|Z}=I(Y_{i+1},X^{i}|Y^{i},Z^{i})\\ \;=KL(p(Y_{i+1}|Y^{i},Z^{i},X^{i});p(Y_{i+1}|Y^{i},Z^{i})), \end{array}$$

which is a conditional mutual information often referred to as transfer entropy (Schreiber, 2000). Analogously, a general information-theoretic measure of instantaneous causality is obtained plugging the probabilities of Equation (6) into Equation (7) (e.g., Rissanen and Wax, 1987; Chicharro and Ledberg, 2012b):

(9) $$\begin{array}{l} T_{X\cdot Y|Z}=I(X_{i+1};Y_{i+i}|X^{i},Y^{i},Z^{i})\\ \;=KL(p(Y_{i+1}|X_{i+1},X^{i},Y^{i},Z^{i});\text{​}p(Y_{i+1}|X^{i},Y^{i},Z^{i})). \end{array}$$

Note that here we use Granger causality to refer to the criterion of conditional independence of Equation (5), and not to the particular measure resulting from its linear implementation (Bressler and Seth, 2011). In that sense, we include in the Granger causality methodology not only the transfer entropy but also other measures developed for example to study causality in the spectral domain (Chicharro, 2011, 2014a).

### Graphical representations of causal interactions

**Figure 4:**
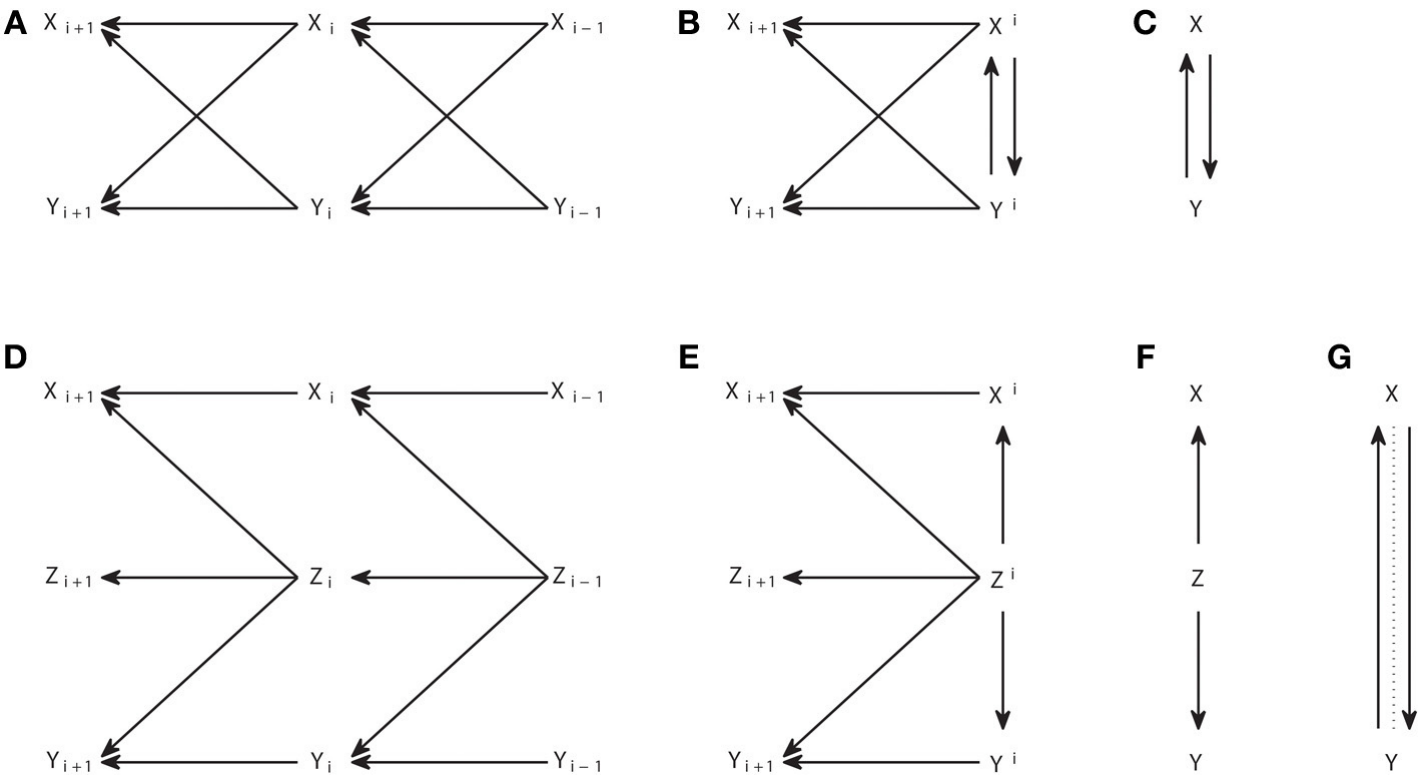
**Graphical representations of interacting processes at different scales. (A–C)** Represent the same bivariate process at a micro, meso, and macroscopic scale. **(D–F)** Represent another process also at these different scales, and **(G)** represents the Granger causal and instantaneous causality relations when only *X* and *Y* are included in the Granger causality analysis.

Causal representations are also commonly used when applying Granger causality analysis. However, we should distinguish other types of causal graphs from the *causal structures*. The connections in a causal structure are such that they reflect in a unique way the arguments of the functions in the causal model which provides a mechanistic explanation of the generation of the variables. This means that, for processes, when the functional model consists of differential equations that in their discretized form are like in Equation (3), the causal structure comprises the variables corresponding to all sampling times, explicitly reflecting the temporal nature of the processes. Figures 4A,D show two examples of interacting processes, the first with two bidirectionally connected processes and the second with two processes driven by a common driver.

The corresponding causal structures constitute a *microscopic* representation of the processes and their interactions, since they contain the detailed temporal information of the exact lags at which the causal interactions occur. However, when many processes are considered together, like in a brain connectivity network, this representation becomes unmanageable. Chicharro and Ledberg (2012b) showed that an intermediate *mesoscopic* representation is naturally compatible with Granger causal analysis, since it contains the same groups of variables used in Equations (5, 6). These graphs are analogous to the *augmentation graphs* used in Dahlhaus and Eichler (2003). At the mesoscopic scale the detailed information of the lags of the interactions is lost and thus also is lost the mapping to the parental structure in the causal model, so that an arrow cannot be associated with a particular causal mechanism. Accordingly, the mesoscopic graphs are not in general DAGs, as illustrated by Figure 4B.

*Macroscopic* graphs offer an even more schematized representation (Figures 4C,F) where each process corresponds to a single node. Moreover, the meaning of the arrows changes depending on the use given to the graph. If one is representing some known dynamics, for example when studying some simulated system, then the macroscopic graph can be just a summary of the microscopic one. On the other hand, for experimental data, the graph can be a summary of the Granger causality analysis and then the arrows represent the connections for which the measure of Granger causality, e.g., the transfer entropy, gives a non-zero value. Analogously, Granger instantaneous causality relations estimated as significant can be represented in the graphs with some undirected link. For example, Figure 4F summarizes the Granger causal relations of the system {*X*, *Y*, *Z*} when all variables are observed, and Figure 4G is a summary of the Granger causal relations (including instantaneous), when the analysis is restricted to the system {*X*, *Y*}, taking Z as a latent process. In Figure 4G the instantaneous causality is indicated by an undirected dotted edge. *Mixed graphs* of this kind have been studied to represent Granger causality analysis, e.g., Eichler (2005, 2007). Furthermore, graph analysis with macroscopic graphs is also common to study structural or functional connectivity (Bullmore and Sporns, 2009).

Apart from the correspondence to a causal model, which is specific of causal structures, it is important to determine for the other graphical representations if it is possible to still apply d-separation or an analogous criterion to read conditional independencies present in the associated probability distributions. Without such a criterion the graphs are only a basic sketch to gain some intuition about the interactions. For mesoscopic graphs, a criterion to derive Granger causal relations from the graph was proposed by Dahlhaus and Eichler (2003) using moralization (Lauritzen, 1996). Similarly, a criterion of separation was proposed in Eichler (2005) for the mixed graphs representing Granger causality and instantaneous Granger causality. However, in both cases these criteria provide only a sufficient condition to identify independencies, even if stability is assumed, in contrast to d-separation for causal structures or patterns, which under stability provides an if and only if condition.

## Extension of pearl’s causal models to dynamic systems and relevance to studying the brain’s effective connectivity

Above we have reviewed two different approaches to causal inference. The approach by Pearl is based on causal models and explicitly considers the limitations of causal inference, introducing the notion of observational equivalence and explicitly addressing the consequences of potential latent variables in the algorithm IC^*^. Conversely, Granger causality more operationally provides a criterion of causality between processes specific for a context, and does not explicitly handle latent influences. Moreover, the Pearl’s approach is not restricted with respect to the nature of the variables and should thus be applicable also to processes. Since this approach is more powerful in how it treats latent variables and in how it indicates the limits of what can be learned, in the following we investigate how the IC and IC^*^ algorithms can be applied to dynamic processes and how they are related to Granger causality.

### Causal inference without latent variables for dynamic processes

We here reconsider the IC algorithm for the especial case of dynamic processes. Of course one can apply the IC algorithm directly, since there are no assumptions about the nature of the variables. However, the causal structures associated with dynamic processes (e.g., the microscopic graphs in Figures 4A,D) have a particular structure which can be used to simplify the algorithm. In particular, the temporal nature of causality assures that all the arrows should point from a variable at time *i* to another at time *i* + *d*, with *d* > 0. This means that the arrows can only have one possible direction. Therefore, once Step 1 has been applied to identify the skeleton of the pattern, all the edges can be assigned a head directly, without necessity to apply Steps 2 and 3. Furthermore, even Step 1 can be simplified, since the temporal precedence give us information of which variables should be used to search for an appropriate set *S_ab_* that renders *a* and *b* conditionally independent. In particular, for *V*_*j,i*_ and *V*_*j*′,*i*+*d*_, indicating the variable of process *j* at the time instant *i* and the variable of process *j*′ at time *i* + *d*, respectively, the existence of *V*_*j,i*_ → *V*_*j*′,*i*+*d*_ can be inferred testing if it does not hold

(10) $$p(V_{j^{\prime },i+d}|{\left\{V\right\}}^{i+d-1})=p(V_{j^{\prime },i+d}|{\left\{V\right\}}^{i+d-1}\backslash V_{j,i}),$$

where {*V*}^*i*+*d*−1^\*V_j,i_* means the whole past of the system at time *i* + *d* excluding *V*_*j,i*_. This is because conditioning on the rest of the past blocks any path that can link the two nodes except a direct arrow. Therefore, *S*_*ab*_ = {*V*}^*i*+*d*−1^\*V_j,i_* is always a valid set to check if *V*_*j,i*_ and *V*_*j*′,*i*+*d*_ are conditionally independent, even if considerations about the estimation of the probability distributions lead to seek for smaller sets (e.g., Faes et al., 2011; Marinazzo et al., 2012).

Note that the combination of the assumption of no latent variables with the use of temporal precedence to add the direction of the arrows straightforwardly after Step 1 of the IC algorithm leads to patterns that are always complete DAGs. This straightforward completion indicates that there is a unique relation between the pattern and the underlying causal structure, that is, there are no two different causal structures sharing the same pattern. For example, from the three causal structures that are observationally equivalent in Figures 1A–C, if only one direction of the arrows is allowed (from right to left for consistency with Figure 4) then only the causal structure of Figure 1B is possible.

There is a clear similarity between the criterion of Equation (10) to infer the existence of a single link in the causal structure and the criterion of Granger causality in Equation (5). In particular, Equation (10) is converted into Equation (5) by two substitutions: (i) taking *d* = 1 and (ii) taking the whole past *V*^*i*+*d*−1^_*j*_ instead of a single node *V*_*j,i*_. Both substitutions reflect that Granger causality analysis does not care about the exact lag of the causal interactions. It allows representing the interactions in a mesoscopic or macroscopic graph, but is not enough to recover the detailed causal structure. By taking *d* = 1 and taking the whole past one is including any possible node that can have a causal influence from process *j* to process *j*′. The Granger causality criterion combines in a single criterion the pile of criteria of Equation (10) for different *d*. Accordingly, in the absence of latent variables, Granger causality can be considered as a particular application of the IC algorithm, simplified accordingly to the objectives of characterizing the causal relations between the processes. Note that this equivalence relies on the stochastic nature of the endogenous variables in Pearl’s model (Equation 1). Furthermore, it is consistent with the relation between Granger causality and notions of structural causality as discussed in White and Lu (2010).

### Causal inference with latent variables for dynamic processes

We have shown above that in the absence of latent processes adding temporal precedence as a constraint tremendously simplifies the IC algorithm and creates a unique mapping between causal structures and patterns. Adding temporal precedence makes causal inference much easier because time provides us with extra information and, in the absence of latent variables, no complications are added when dealing with dynamic processes.

**Figure 5:**
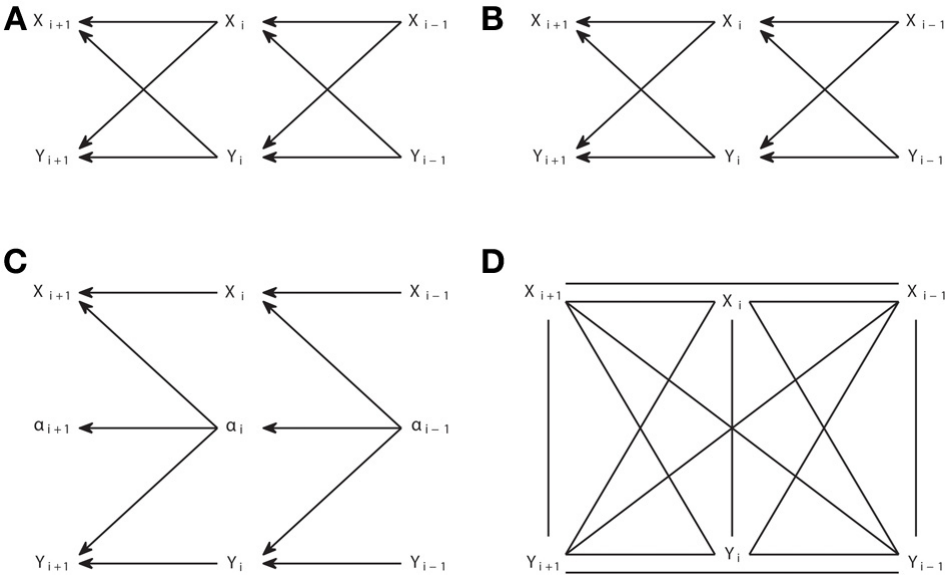
Causal structures corresponding to interacting dynamic processes (A,C) and their corresponding embedded patterns retrieved from the IC^*^ algorithm (B,D).

We now show that this simplification does not hold anymore when one considers the existence of latent processes. We start with two examples in Figure 5 that illustrate how powerful or limited can be the application of the IC^*^ algorithm to dynamic processes. Note that the IC^*^ algorithm is applied taking the causal structures in Figures 5A,C as an interval of stationary processes, so that the same structure holds before and after the nodes displayed.

In Figure 5A we display a causal structure of two interacting processes without any latent process, and in Figure 5B the corresponding embedded pattern. We can see that, even allowing for the existence of latent processes, the IC^*^ algorithm can result in a DAG which completely retrieves the underlying causal structure. In this case the output of the IC algorithm and of the IC^*^ algorithm are the same pattern, but the output of the IC^*^ algorithm is actually a much stronger result, since it states that a bidirectional genuine causation must exist between the processes even if one considers that some other latent processes exist.

Conversely, consider the causal structure of Figure 5C in which *X* and *Y* are driven by a hidden process. The resulting embedded pattern is a completely filled undirected graph, in which all nodes are connected to all nodes since there are no conditional independencies. Further using the extra information provided by temporal precedence—by substituting all horizontal undirected links by dashed arrows pointing to the left and vertical links by bidirectional arrows—does not allow us to better retrieve the underlying causal structure since, unlike the patterns resulting from the IC algorithm, the embedded patterns resulting from the IC^*^ algorithm do not have to share the skeleton with the causal structures belonging to their dependency equivalence class.

The IC^*^ algorithm is not suited to study dynamic processes for two main reasons. First, the embedded pattern chosen as a representation of the dependency class is strongly determined by the selection of projections as the representative subset of the class. The projections exclude connections between the latent variables or latent variables connected to more than two observed variables. By contrast, a latent process generally consists *per se* in a complex structure of latent variables. In particular, commonly causal interactions exist between the latent nodes, since most latent processes will have a causal dependence on their own past, and each node does not have a causal influence on only two observable nodes.

Second, the IC^*^ algorithm is designed to infer the causal structure associated with the causal model. This means that, for dynamic processes, for which generally an acyclic directed graph is only obtained when explicitly considering the dynamics, the IC^*^ algorithm necessarily infers the microscopic representation of the causal interactions. In contrast to the case of the IC algorithm in which there are no latent variables, it is not possible to establish an immediate correspondence with Granger causality analogous to the relation between Equation (5) and Equation (10). The fact that the IC^*^ algorithm necessarily has to infer the microscopic causal structure is not desirable for dynamic processes. This is because of several reasons related to the necessity to handle a much higher number of variables (nodes). In first instance, it requires the estimation of many more conditional independencies in Step 1 of the algorithm, which is a challenge for practical implementations (see Supplementary Material for discussion of the implementation of the algorithms). In second instance, the microscopic embedded pattern, as for example the one in Figure 5D, can be too detailed without actually adding any information about the underlying causal structure but, on the contrary, rendering the reading of its basic structure less direct.

Here we propose a new algorithm to obtain a representation of the dependency class when studying dynamic processes. The new algorithm recasts the basic principles of the IC^*^ algorithm but has the advantage that it avoids the assumptions related to the projections, and allows to study causal interactions between the processes at a macroscopic level, without necessarily examining the lag structure of the causal interactions. With respect to usual applications of Granger causality, the new algorithm has the advantage that it explicitly considers the existence of potential latent processes. It is important to note that the new algorithm is not supposed to outperform the IC^*^ algorithm in the inference of the causal interactions. They differ only in the number of conditional independencies that have to be tested, much lower for the new algorithm since only the macroscopic causal structure is examined, and in the form of the embedded pattern chosen to represent the dependency equivalent class. In simpler terms, for dynamic processes, the new algorithm offers a more appropriate representation of the class of networks compatible with the estimated conditional independencies. Both algorithms rely on the same framework to infer causality from conditional independencies, and theoretically their performance is only bounded by the existence of observationally equivalent causal structures. None of the two algorithms addresses the practical estimation of the conditional independencies, and thus any evaluation of their practical performance is specific to the particular choice of how to test conditional independence (see Supplementary Material for discussion of the implementation).

In comparison to the assumptions related to projections, the new algorithm assumes that any latent process is such that its present state depends in a direct causal way on its own past, that is, that its autocorrelation is not only indirectly produced by the influence of other processes. In practice, this means that we are excluding cases like an uncorrelated white noise that is a common driver of two observable processes. The reason for this assumption is that, excluding these processes without auto-causal interactions, we have (Chicharro and Ledberg, 2012b) that there is a clear difference between the effect of hidden common drivers and the effect of hidden processes that produce indirect causal connections (i.e., *X* → α → *Y*). In particular, if we have a system composed by two observable processes *X* and *Y* such that a hidden process α mediates the causal influence from *X* to *Y*, we have that

(11) $$X\to \alpha \to Y\Rightarrow T_{X\to Y}>0\wedge T_{X\cdot Y}=0,$$

where ∧ indicates conjunction. Conversely, if the system α is a common driver we have that

(12) $$X\leftarrow \alpha \to Y\Rightarrow T_{X\to Y}>0\wedge T_{X\cdot Y}>0,$$

We see that common drivers and mediators have a different effect regarding the induction of instantaneous causality. This difference generalizes to multivariate systems with any number of observed or latent processes (see Supplementary Material). Common drivers are responsible for instantaneous causality. In fact, if there is no set of observable processes such that when conditioning on it the instantaneous causality is canceled, then some latent common drivers must exist since *per se* causality cannot be instantaneous unless we think about entanglement of quantum states. Accordingly,

(13) $$\begin{array}{l} \forall ST_{X\cdot Y|S}>0\Leftrightarrow \text{common driver latent processes cause}\\ \text{ instantaneous causality}, \end{array}$$

where one or more common driver latent processes may be involved. Properties in Equations (11–13) are used in the new algorithm. The input is the joint distribution that includes the variables corresponding to sampling time *i* + 1 and to the past of the observable processes *V*_*O*_, i.e., *p*({*V*_*Oi*+1_}, {*V^i^_O_*}). The output is a macroscopic graph which reflects all and no more Granger causality and instantaneous causality relationships than the ones present in *p*({*V*_*Oi*+1_}, {*V^i^_O_*}). The algorithm proceeds as follows:

### ICG^*^ algorithm (inductive causation with latent variables using granger causality)

1. For each pair of processes *a* and *b* in {*V_O_*} search for a set *S_ab_* of processes such that *T_a·b|S_ab__* = 0 holds in *p*({*V_O_*}), i.e., there is no instantaneous causality between *a* and *b* given *S_ab_*. Construct a macroscopic graph with each process represented by one node and linking the nodes *a* and *b* with a bidirectional arrow *a* ↔ *b* if and only if *S_ab_* is not found.
2. For each pair *a* and *b* not linked by a bidirectional arrow search for a set *S_ab_* of processes such that *T*_*a*→*b*|*S_ab_*_ = 0 holds in *p*({*V_O_*}), i.e., there is no Granger causality from *a* to *b* given *S_ab_*. Link the nodes *a* and *b* with a unidirectional arrow *a*→*b* if and only if *S_ab_* is not found.
3. For each pair *a* and *b* not linked by a bidirectional arrow search for a set *S_ab_* of processes such that *T*_*b*→*a*|*S_ab_*_ = 0 holds in *p*({*V_O_*}), i.e., there is no Granger causality from *b* to *a* given *S*_*ab*_. Link the nodes *a* and *b* with a unidirectional arrow *a* ← *b* if and only if *S_ab_* is not found.

The zero values of the Granger measures indicate the existence of some conditional independencies. Step 1 identifies the existence of latent common drivers whenever Granger instantaneous causality exists and marks it with a bidirectional arrow. Steps 2 and 3 identify Granger causality in each direction when there is no Granger instantaneous causality. In fact Granger causality will also be present for the bidirectionally linked nodes, but there is no need to check it separately, given Equation (12). Steps 1–3 are analogous to Step 1 of the IC^*^ algorithm since conditioning sets of different size have to be screened, but now the conditional independencies examined are not between single variables but between processes and this is why Granger causality measures are used.

The algorithm differs in two principle ways from how Granger causality is commonly used. First, Granger causality is not applied once for each pair of nodes, but one has to search for a context that allows assessing if a conditional independence exists. This is different from applying bidirectional Granger causality to all combinations of nodes, and also from applying to all combinations of nodes conditional Granger causality conditioning on the whole rest of the system. The reason is that, as discussed in Hsiao (1982) and Ramb et al. (2013), when latent processes exist, further adding new processes to the conditioning can convert a zero Granger causality into positive.

Second, an explicit consideration of the possible existence of latent processes is incorporated, to our knowledge for the first time, when applying Granger causality. A bidirectional arrow indicates that the dependencies between the processes can only be explained by latent common drivers. We should note that this does not discard that in addition to common drivers there are directed causal links between the processes, in the same way that unidirectional arrows do not discard that the causal influence is not direct but through a mediator latent processes. This is because the output of the algorithm is again a representation of a class of causal structures and thus these limitations are common to the IC^*^ algorithm which also implicitly allows the existence of multiple hidden paths between two nodes or of latent mediators. Of course, when studying brain connectivity it can be relevant to establish for example if two regions are directly causally connected, but this cannot be done without recording from the potential intermediate regions, or using some heuristic knowledge of the anatomical connectivity.

The output of the ICG^*^ algorithm most often is more intuitive about the causal influences between the processes than the embedded pattern resulting from the IC^*^ algorithm and does not need to consider the microscopic structure. For example, while for the causal structure of Figure 5C we found that the IC^*^ algorithm provides as output the embedded pattern of Figure 5D (which has a lot of edges that are not in the underlying causal structure so that a direct mapping is not possible), we found that the ICG^*^ algorithm simply provides as output *X* ↔ *Y* thereby revealing synthetically, directly, and correctly the existence of at least one latent common driver.

However, to be meaningful as a representation of the conditional independencies associated with the Granger causality relationships, we need to complement the algorithm with a criterion of separation analogous to the one available for the patterns and embedded patterns obtained from the IC and IC^*^ algorithms, respectively. In particular, d-separation can be again used, now considering a collider on a path to be any node with two head to head arrows on the path, where the heads can belong to the two types of arrows, i.e., unidirectional or bidirectional. Accordingly, the subsequent sufficient conditions can be applied to read the Granger causal relations from the graph:

**Graphical sufficient condition for Granger non-causality**
*X* is d-separated from *Y* by *S* on each path between *X* and *Y* with an arrow pointing to *Y* ⇒ *T*_*X* → *Y*|*S*_ = 0.
**Graphical sufficient condition for instantaneous non-causality**
*X* is d-separated from *Y* by *S* on each path between *X* and *Y* with an arrow pointing to *X* and an arrow pointing to *Y* ⇒ *T*_*X* · *Y*|*S*_ = 0.

Proofs for these conditions are provided in the Supplementary Material. As in general for d-separation, these conditions become if and only if conditions if further assuming *stability*. The conditions here introduced for the graphs resulting from the ICG^*^ algorithm are very similar to the ones proposed by Eichler (2005) for mixed graphs. Also for mixed graphs Eichler (2009) proposed an algorithm of identification of Granger causality relationships. The critical difference with respect to this previous approach is that here instantaneous causality is considered explicitly as the result of existing latent variables, according to Equations (11–13), while in the mixed graphs there is no explanation of how it arises from the underlying dynamics.

### Analysis of the effect of latent variables

The results above concern the application of general algorithms of causal inference to dynamic processes, and how these algorithms are related to the Granger causality analysis. The perspective was focused on how to learn the properties of an unknown causal structure from the conditional independencies contained in a probability distribution obtained from recorded data. In this section we address the opposite perspective, i.e., we assume that we know a causal structure and we focus on examining what we learn by reading the conditional independencies that are present in any distribution compatible with the structure. We will see that a simple analysis applying d-separation can explain in a simple way many of the scenarios in which Granger causality analysis can lead to inconsistent results about the causal connections. We here term the positive values of Granger causality that do not correspond to arrows in the causal structure as *inconsistent positives*. These are to be distinguished from *false positive*s as commonly understood in hypothesis testing, since the inconsistent positives do not result from errors related to estimation, but, as we show below, they result from the selection of subordinate signals as the ones used to carry out the causal inference analysis.

The definition of d-separation does not provide a procedure to check if all paths between the two variables which conditional independence is under consideration have been examined. However, a procedure based on graphical manipulation exists that allows checking all the paths simultaneously (Pearl, 1988; Kramers, 1998). We here illustrate this procedure to see how it supports the validity of Granger causality for causal inference when there are no latent processes and then apply it to gain more intuition about different scenarios in which inconsistent positive values are obtained. The procedure works as follows: to check if *X* is d-separated from *Y* by a set *S*, first create a subgraph of the complete structure including only the nodes and arrows that are attained moving backward from *X*, *Y* or the nodes in *S* (i.e., only the ancestors an(*X*,*Y*,*S*) appear in the subgraph); second, delete all the arrows coming out of the nodes belonging to S; finally, check if there is still any path connecting *X* and *Y* and if such a path does not exist, *X* and *Y* are separated by *S*.

**Figure 6:**
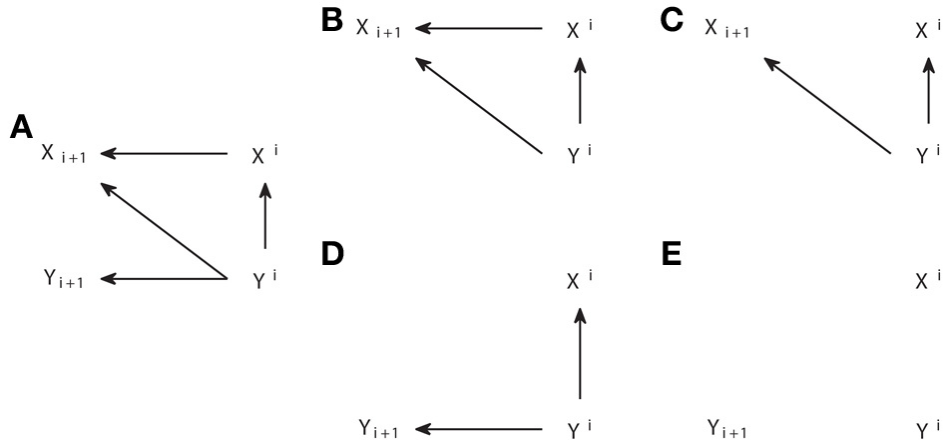
**Graphical procedure to apply d-separation to check the conditional independencies associated with Granger causality. (A)** Causal structure corresponding to a system with unidirectional causal connections from process *Y* to *X*. **(B,C)** Steps 1 and 2 of modification of the original graph in order to check if *T*_*Y* → *X*_ = 0. **(D,E)** Analogous to **(B,C)**, but to check if *T*_*X* → *Y*_ = 0.

In Figure 6 we display the modifications of the graph performed to examine the conditional independencies associated with the criterion of Granger causality. In Figure 6A we show the mesoscopic graph of a system with unidirectional causal interactions from *Y* to *X*. In Figures 6B,C we show the two subsequent modifications of the graph required to check if *T*_*Y* → *X*_ = 0, while in Figures 6D,E we show the ones required to check if *T*_*X* → *Y*_ = 0. In Figure 6B the subgraph is selected moving backward from {*X*_*i*+1_, *X^i^*, *Y^i^*}, the nodes involved in the corresponding criterion in Equation (5). In Figure 6C the arrow leaving the conditioning variable *X*^*i*^ is removed. The analogous procedure is followed in Figures 6D,E. It can be seen that in Figure 6C *Y*^*i*^ and *X*_*i*+1_ are still linked, indicating that *T*_*Y* → *X*_ > 0, while there is no link between *X*^*i*^ and *Y*_*i*+1_ in Figure 6E, indicating that *T*_*X* → *Y*_ = 0.

**Figure 7:**
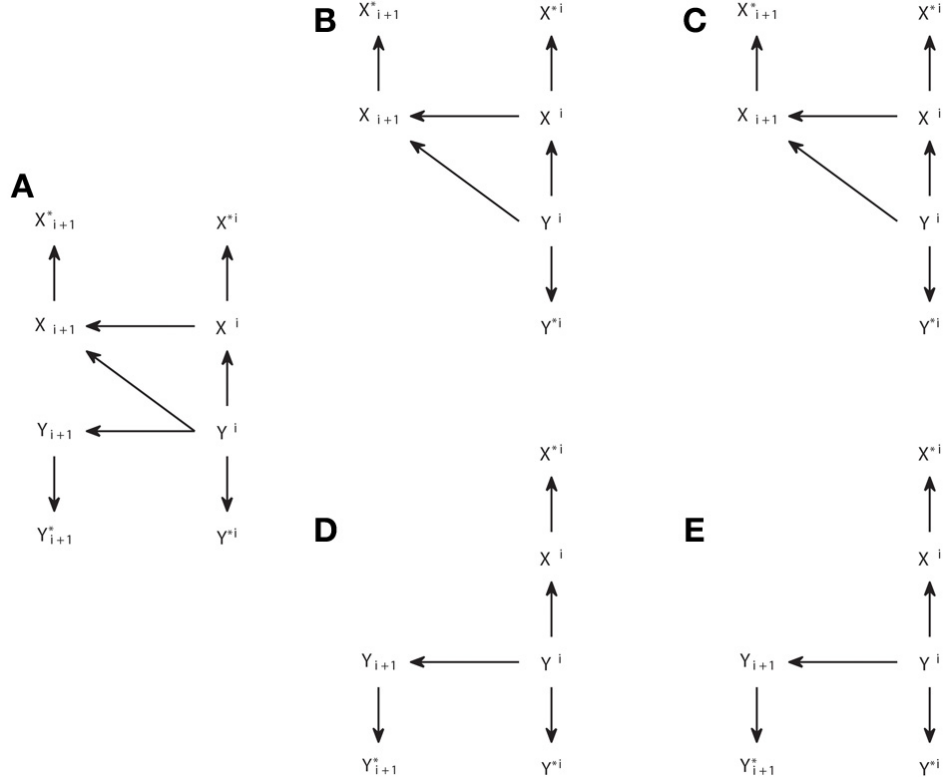
Analogous to Figure 6 but for a causal structure in which the subordinate processes X^*^ and Y^*^ are recorded instead of processes *X* and *Y* between which the causal interactions occur. (A) The original causal structure. **(B,C)** Steps 1 and 2 of modification in order to check if *T*_*Y* → *X*_ = 0. **(D,E)** analogous to **(B,C)**, but to check if *T*_*X* → *Y*_ = 0.

Therefore, d-separation allows us to read the Granger causal relations from the structure of Figure 6A. One may ask why we should care about d-separation providing us with information which is already apparent from the original causal structure in Figure 6A that we assume to know. The answer is that, when one constructs a causal structure to reproduce the setup in which the observable data are recorded, the Granger causal relations between those are generally not so obvious from the causal structure. To illustrate that, we consider below a quite general case in which the Granger causality analysis is not applied to the actual processes between which the causal interactions occur, but to some time series derived from them. In Figure 7A we display the same system with a unidirectional causal interaction from *Y* to *X*, but now adding the extra processes X^*^ and Y^*^, which are obtained by some processing of *X* and *Y*, respectively. If only the processes X^*^ and Y^*^ are observable, and the Granger causality analysis is applied to them, this case comprises scenarios such as the existence of measurement noise, or the case of fMRI in which the observed BOLD responses only indirectly reflect the hidden neuronal states (Friston et al., 2003; Seth et al., 2013).

We can see in Figure 7C that *T*_Y^*^ → X^*^_ > 0, as if the analysis was done on the original underlying processes *X* and *Y*, for which *T*_*Y* → *X*_ > 0. However, in the opposite direction we see in Figure 7E that an inconsistent positive value appears, since also *T*_X^*^ → Y^*^_ > 0, while *T*_*X* → *Y*_ = 0. We can see that this happens because *Y*^*i*^ acts as a common driver of Y^*^_i+1_ and X^*i^, through the paths *Y*^*i*^ → *Y*_*i*+1_→ Y^*^_i+1_ and *Y*^*i*^ → *X^i^* → X^*i^, respectively. This case, in which the existence of a causal interaction in one direction leads to an inconsistent positive in the opposite direction when there is an imperfect observation of the driven system (here Y), has been recently discussed in Smirnov (2013). Smirnov (2013) has exemplified that the effect of measurement noise or time aggregation—due to low sampling- can be understood in this way. However, the illustration in Smirnov (2013) is based on the construction of particular examples and requires complicated calculations to obtain analytically the Granger causality values. With our approach, general conclusions are obtained more easily by applying d-separation to a causal structure that correctly captures how the data analyzed are obtained. Nonetheless, the use of graphical criteria and exemplary simulations is complementary, since one advantage of the examples in Smirnov (2013) is that it is shown that the non-negative values of the Granger causality measure in the opposite direction can have a magnitude comparable or even bigger than those in the correct direction.

In Table 1 we summarize some paradigmatic common scenarios in which a latent process acts as a common driver leading to inconsistent positives in Granger causality analysis. In all these cases Granger causality can easily be assessed in a general way from the corresponding causal structure that includes the latent process. First, when non-stationarities exist, time can act as a common driver since the time instant provides information about the actual common dynamics. This is the case for example of cointegrated processes, for which an adapted formulation of Granger causality has been proposed (Lütkepohl, 2005). Also event-related setups may produce a common driver, since the changes in the ongoing state from trial to trial can simultaneously affect the two processes (e.g., Wang et al., 2008).

**Table 1:** **Cases in which a hidden common driver leads to inconsistent positive Granger causality from the observed process derived from process ***X*** to the observed process derived from process *Y* when there are unidirectional causal connections from ***Y*** to *X* (or processes ***Y_k_*** to *X_k_*)**.

		**Observed variables**	**Common driver**
1	Non-stationarity	*X_i_* and *Y_i_*	Time
2	Event-related setup	*X_i_* and *Y_i_*	Trial ongoing state
3	Discretizing	Bin(*X*)_*i*_ and Bin(*Y*)_*i*_	Underlying process *Y*
4	Measurement noise	X_i_^*^ = *X_i_* + ε_*x,i*_ and Y_i_^*^ = *Y_i_* + ε_*y,i*_	Underlying process *Y*
5	fMRI analysis	h(*X*)_*i*_ and h(*Y*)_*i*_	Underlying process *Y*
6	Time aggregation	*X*_*Ti*_ and *Y*_*Ti*_	Unsampled time instants of *Y*
7	Spatial aggregation	X^*^_i_ = Σ*_k_X_k,i_* and Y^*^_i_ = Σ_*k*_ *Y*_*k,i*_	Underlying processes *Y_k_*

The other cases listed in Table 1 are analogous to the one illustrated in Figure 7. Discretizing continuous signals can induce inconsistent positives (e.g., Kaiser and Schreiber, 2002) and also measurement noise (e.g., Nalatore et al., 2007). In both cases Granger causality is calculated from subordinate signals, obtained after binning or after noise contamination, which constitute a voluntary or unavoidable processing of the underlying interacting processes. Similarly, the hemodynamic responses h(*X*) and h(*Y*) only provide with a subordinate processed signal from the neural states (e.g., Roebroeck et al., 2005; Deshpande et al., 2010). In the case of time aggregation, the variables corresponding to unsampled time instants are the ones acting as common drivers (Granger, 1963). The continuous temporal nature of the processes has been indicated as a strong reason to advocate for the use of DCM instead of autoregressive modeling (see Valdes-Sosa et al., 2011 for discussion). Finally, aggregation also takes place in the spatial domain. To our knowledge, the consequences of spatial aggregation for the interpretation of the causal interactions have been studied less extensively so far than those posed by time aggregation, and thus we focus on spatial aggregation in the section below.

### The case of spatial aggregation

We next investigate what happens when it is not possible to measure directly the activity of the neural sources among which the causal interactions occur because only spatially aggregated signals that aggregate many different neural sources are recorded. For example, a single fMRI voxel reflects the activity of thousands of neurons (Logothetis, 2008), or the local Field Potential amplitude measured at a cortical location captures contributions from several sources spread over several hundreds of microns (Einevoll et al., 2013). The effect of spatial aggregation on stimulus coding and information representations has been studied theoretically (Scannell and Young, 1999; Nevado et al., 2004), but its effect on causal measures of the kind considered here still needs to be understood in detail.

Possible distortions introduced by spatial aggregation depend on the nature of the processes and the scale at which the analysis is done. In particular, neuronal causal interactions occur at a much more detailed scale (e.g., at the level of synapses) than the scale corresponding to the signals commonly analyzed. It is not clear, and to our knowledge it has not been addressed, how causal relations at a detailed scale are preserved or not when zooming out to a more macroscopic representation of the system. As we will discuss in more depth in the Discussion, the fact that a macroscopic model provides a good representation of macroscopic variables derived from the dynamics does not assure that it also provides a good understanding of the causal interactions.

In general, the effect of spatial aggregation on causal inference can be understood examining a causal structure analogous to the one of Figure 7, but where instead of a single pair of underlying processes *X* and *Y* there are two sets *X_k_*, *k* = 1, …, *N*, and *Y*_*k*′_, *k*′ = 1, …, *N*′ between which the causal interactions occur. The signals observed are just an average or a sum of the processes, $X^{*}=\sum_{k=1}^{N}X_{k}\text{ and }Y^{*}=\sum_{k=1}^{N^{\prime }}Y_{k}$. For example, in the case of the brain, the processes can correspond to the firing activity of individual neurons, and the recorded signals to some measure of the global activity of a region, like the global rates *r*_*X*_ and *r_y_*. Even if for each pair *X_k_*, *Y_k_* a unidirectional causal connection exists, the Granger causality between *r*_*X*_ and *r_y_* will be positive in both directions, as can be understood from Figure 7.

We will now examine some examples of spatial aggregation. As we mentioned in the Introduction, here we specifically focus on causal inference, i.e., determining which causal interactions exist. We do not address the issue of further quantifying the magnitude of causal effects, since this is generally more difficult (Chicharro and Ledberg, 2012b; Chicharro, 2014b) or even in some cases not meaningful (Chicharro and Ledberg, 2012a). In the case of spatial aggregation, the fact that Granger causality calculated from the recorded signals has always positive values in both directions is predicted by the graphical analysis based on d-separation. However, in practice the conditional independencies have to be tested from data instead of derived using Equation (4). When tested with Granger causality measures, the magnitude of the measure is relevant, even if not considered as a quantification of the strength of the causal effect, because it can determine the significance of a non-negative value. The relation between magnitude and significance depends on the estimation procedure and on the particular procedure used to assess the significance levels (e.g., Roebroeck et al., 2005; Besserve et al., 2010). It is not on the focus of this work to address a specific implementation of the algorithms of causal inference, which requires specifying these procedures (see Supplementary Material for discussion). Nonetheless, we now provide some numerical examples following the work of Smirnov (2013) to illustrate the impact of spatial aggregation on the magnitude of the Granger causality measures and we show that the inconsistent positives can have comparable or even higher magnitude than the consistent positives, and thus are expected to impair the causal inference performance.

**Figure 8:**
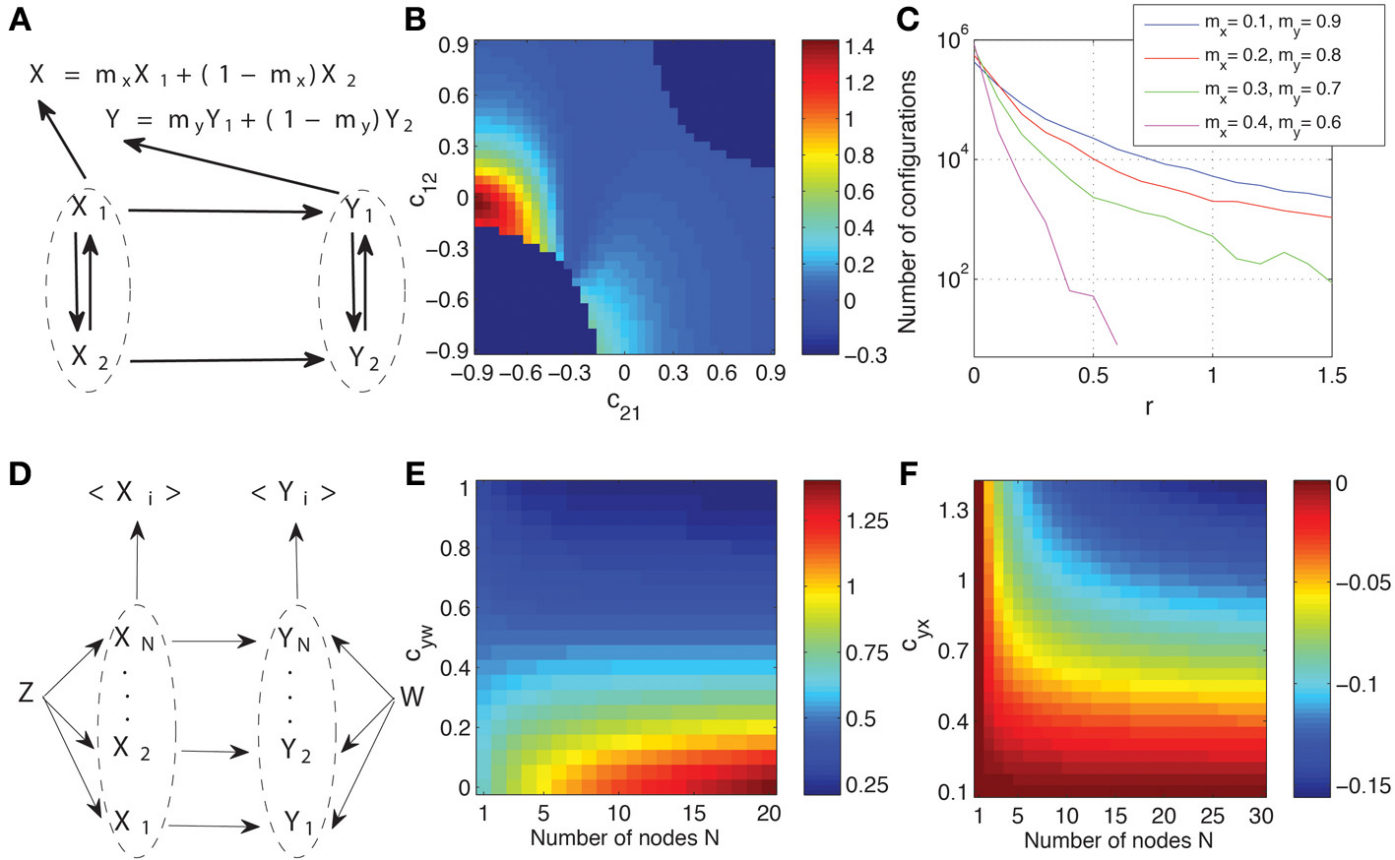
**Effects of spatial aggregation on Granger causality. (A)** Causal graph representing two areas composed each of two processes and from which signals are recorded as a weighted sum. See the text for details of how a system compatible with the graph is generated as a multivariate linear Gaussian autoregressive process. **(B)** Dependence of the relative magnitude *r* of the inconsistent positives of Granger causality (Equation 15) on the space formed by coupling coefficients between *X*_1_ and *X*_2_. **(C)** Number of configurations with a given value *r* for all stationary configurations in the space of the parameters *c*_11_, *c*_22_, *c*_12_, and *c*_21_ and for different weights combinations. **(D)** Another example of causal graph where spatial aggregation is present in the recording of the signals from the two areas. The system is again generated as a multivariate autoregressive process with identical connections from *Z* to each *X_k_*, identical from *W* to each *Y_k_*, and identical from each *X_k_* to each *Y_k_* (see the main text for details). **(E)** The Granger causality measure *T*_<*X*>→<*Y*>_ as a function of the coefficient *c*_*yw*_ and the number of processes *N*. **(F)** The relative changes Δ*T*′ (Equation 16) of the Granger causality measure as a function of the coefficient *c*_*yx*_ and the number of processes *N*.

In Figure 8A we show the macroscopic graph representing the spatial aggregation of two processes in two areas, respectively. The processes are paired, so that a unidirectional interaction from *X_k_* to *Y_k_* exists, but the signals recorded on each area are a weighted sum of the processes, that is, we have *X* = *m_x_X*_1_ + (1 − *m_x_*) *X*_2_, and analogously for *Y* with *m_y_*. This setup reproduces some basic properties of neural recordings, in which different sources contribute with different intensity to the signal recorded in a position. To be able to calculate analytically the Granger causality measures we take, as a functional model compatible with the causal structure that corresponds to Figure 8A, a multivariate linear Gaussian autoregressive process. Considering the whole dynamic process W = {*X*_1_, *X*_2_, *Y*_1_, *Y*_2_}, the autoregressive process is expressed as

(14) $$\left(\begin{array}{c} X_{1i+1}\\ X_{2i+1}\\ Y_{1i+1}\\ Y_{2i+1} \end{array}\right)=\left(\begin{array}{cccc} c_{11} & c_{12} & 0 & 0\\ c_{21} & c_{22} & 0 & 0\\ 0.8 & 0 & 0.8 & 0\\ 0 & 0.8 & 0 & 0.8 \end{array}\right)\left(\begin{array}{c} X_{1i}\\ X_{2i}\\ Y_{1i}\\ Y_{2i} \end{array}\right)+\left(\begin{array}{c} \varepsilon_{x1i}\\ \varepsilon_{x2i}\\ \varepsilon_{y1i}\\ \varepsilon_{y2i} \end{array}\right),$$

where *C* is the matrix that determines the connectivity. For example, the coefficient *c*_12_ indicates the coupling from *X*_2_ to *X*_1_. Matrix *C* is compatible with the graph of Figure 8A: we fix *c*_13_ = *c*_14_ = *c*_23_ = *c*_24_ = *c*_32_ = *c*_41_ = 0 so that inter-areal connections are unidirectional from *X_k_* to *Y_k_*. Furthermore, to reduce the dimensions of the parameter space to be explored, we also fix *c*_34_ = *c*_43_ = 0, so that *Y*_1_ and *Y*_2_ are not directly connected, and *c*_31_ = *c*_42_ = *c*_33_ = *c*_44_ = 0.8. The autoregressive process is of order one because the future values at time *i* + 1 only depend on time at *i*. We assume that there are no latent influences and thus the different components of the noise term ε are uncorrelated, i.e., the innovations have a diagonal covariance matrix. We fix the variance of all innovations to 1. Accordingly, the parameter space that we explore involves the coefficients *c*_11_, *c*_22_, *c*_12_, and *c*_21_. We exclude those configurations which are non-stationary.

The observed signals are obtained from the dynamics as a weighted average. The Granger causality measures can then be calculated analytically from the second order moments (see Chicharro and Ledberg, 2012b and Smirnov, 2013 for details). In all cases 20 time lags of the past are used, which is enough for convergence. If the Granger causality measures were calculated for each pair of underlying processes separately, we would get always *T*_*X*_*k*_ → *Y*_*k*__ > 0 and *T*_*Y*_*k*_ → *X*_*k*__ = 0. However, for the observed signals *X* and *Y*, inconsistent positive are expected. To evaluate the magnitude of these inconsistent positives we calculate their relative magnitude.

(15) $$r=T_{Y\to X}/T_{X\to Y}.$$

In Figure 8B we show the values of *r* in the space of *c*_12_, *c*_21_, fixing *c*_11_ = 0.8 and *c*_22_ = 0.2. Furthermore, we fix *m_x_* = 0.3 and *m_y_* = 0.7. This means that *X*_2_ has a preeminent contribution to *X* while *Y*_1_ has a preeminent contribution to *Y*. We indicate the excluded regions where non-stationary processes are obtained with *r* = −0.3. In the rest of the space *r* is always positive, but can be low (~10^−5^). However, for some regions *r* is on the order of 1, and even bigger than 1. In particular, this occurs around *c*_12_ = 0, where *T*_*X* → *Y*_ is small, but also around *c*_21_ = 0, where *T*_*X* → *Y*_ is high. Here we only intend to illustrate that non-negligible high values of *r* are often obtained, and we will not discuss in detail why some particular configurations enhance the magnitude of the inconsistent positives (a detailed analysis of the dependencies can be found in Chicharro and Ledberg, 2012b and Smirnov, 2013). In Figure 8C we show the number of configurations in the complete space of the parameters *c*_11_, *c*_22_, *c*_12_, and *c*_21_ in which a given *r*-value is obtained. We show the results for four combinations of weights. We see that the presence of values *r* > 0.1 is robust in this space, and thus it is not only for extreme cases that the inconsistent positives would be judged as having a non-negligible relative magnitude. In particular, for this example, *r* increases when the weights at the two areas differ, consistently with the intuition that the underlying interactions can be characterized worse when processes from different pairs are preeminently recorded in each area. Note that none of the algorithms of causal inference, including in particular the ICG^*^, can avoid obtaining such inconsistent positives. In fact, for the examples of Figure 8, in which the only two analyzed signals are those that are spatially aggregated, the ICG^*^ algorithm is reduced to the calculation of *T*_*X* → *Y*_, *T*_*Y* → *X*_, and *T*_*X.Y*_ for these two signals. This illustrates that no algorithm of causal inference can overcome the limitation of not having access to the sources between which the causal interactions actually occur.

In the example above we focused on evaluating the relative magnitude of inconsistent positives of Granger causality. However, spatial aggregation also affects the magnitude of Granger causality in the direction in which a true underlying causal connection exists. We also examine these effects since, although as we mentioned above it may not be safe to use this magnitude as a measure of the strength of the causal effect, it has been widely used with this purpose or more generally as a measure of directional connectivity (see Bressler and Seth, 2011 for a review). To appreciate this, we examine a system sketched in the macroscopic graph of Figure 8D. Here we consider two areas *X* and *Y* each comprising *N* processes. For simplification, instead of considering causal connections internal to each area, the degree of integration within each area is determined by a common driver to all the processes of one area, *Z* for *X_k_* and *W* for *Y_k_*. The coupling between the areas is unidirectional for the pairs *X_k_* → *Y_k_*, and only the average of all the processes is recorded from each area, <*X*> and <*Y*>. We now focus on examining how *T*_<*X*>→<*Y*>_ depends on the number of processes *N*. Again, the processes are generated with a multivariate autoregressive process for which the entries of the coefficient matrix *C* are compatible with the connections of Figure 8D:

(16) $$\left(\begin{array}{l} X_{1i+1}\\ \vdots \\ X_{Ni+1}\\ Z_{i+1}\\ Y_{1i+1}\\ \vdots \\ Y_{Ni+1}\\ W_{i+1} \end{array}\right)=\left(\begin{array}{cccccccc} c_{xx} & \cdots & 0 & c_{xz} & 0 & \vdots & 0 & 0\\ \vdots & \ddots & \vdots & \vdots & \vdots & \ddots & \vdots & \vdots \\ 0 & \cdots & c_{xx} & c_{xz} & 0 & \cdots & 0 & 0\\ 0 & \cdots & 0 & c_{zz} & 0 & \cdots & 0 & 0\\ c_{yx} & \cdots & 0 & 0 & c_{yy} & \cdots & 0 & c_{yw}\\ \vdots & \ddots & \vdots & \vdots & \vdots & \ddots & \vdots & \vdots \\ 0 & \cdots & c_{yx} & 0 & 0 & \cdots & c_{yy} & c_{yw}\\ 0 & \cdots & 0 & 0 & 0 & \cdots & 0 & c_{ww} \end{array}\right)\left(\begin{array}{l} X_{1i}\\ \vdots \\ X_{Ni}\\ Z_{i}\\ Y_{1i}\\ \vdots \\ Y_{Ni}\\ W_{i} \end{array}\right)\left(\begin{array}{l} \varepsilon_{x1i}\\ \vdots \\ \varepsilon_{xNi}\\ \varepsilon_{zi}\\ \varepsilon_{y1i}\\ \vdots \\ \varepsilon_{yNi}\\ \varepsilon_{wi} \end{array}\right)$$

Furthermore, the innovations covariance matrix is again an identity matrix. In Figure 8E we fix all the non-zero coefficients to 0.8 except *c*_*xz*_ and *c*_*yw*_, which determine the degree of integration in area *X* due to the common driver *Z*, and of area *Y* due to common driver *W*, respectively. We then display *T*_<*X*>→<*Y*>_ as a function of *c*_*yw*_ and *N* fixing *c*_*xz*_ = 0.5, in the middle of the interval [0, 1] examined for *c*_*yw*_. We see that *T*_<*X*>→<*Y*>_ either increases or decreases with *N* depending on which coupling is stronger, *c*_*xz*_ or *c*_*yw*_. This means that, *T*_<*X*>→<*Y*>_, which is commonly interpreted as a measure of the strength of the connectivity between the areas, is highly sensitive to properties internal to each of the region when evaluated at a macroscopic scale at which spatial aggregation is present. Changes in the level of intra-areal integration could be interpreted as changes in the inter-areal interactions, but in fact *T*_*X*_*k*_ → *Y*_*k*__ is constant for all the configurations shown in Figure 8E.

In Figure 8F we examine how vary, depending on the number of processes *N*, the changes of *T*_<*X*>→<*Y*>_ as a function of the actual coupling coefficient between the areas at the lower scale (*c*_*yx*_). We again fix all the non-zero coefficients to 0.8 except *c*_*xz*_ = 1.4, *c*_*xx*_ = 0.2, and *c_yx_* ∈ [0.1, 1.4]. Since *c_xz_* > *c_yw_* the Granger causality increases with *N*. We examine if this increase is different depending on *c_yx_*. For that purpose, for each value of *N* we take as a reference the Granger causality calculated for the lowest coupling *c*_*yx*_ = 0.1. We then calculate *T*′_<*X*>→<*Y*>_(*c_yx_, N*) =*T*_<*X*>→<*Y*>_(*c_yx_, N*)/*T*_<*X*>→<*Y*>_(0.1, *N*), that is, the proportion of the Granger causality for each *c_yx_* with respect to the one for *c*_*yx*_ = 0.1. We then consider the relative changes of *T*′_<*X*>→<*Y*>_(*c_yx_, N*) depending on *N*:

(17) $$\Delta T^{\prime }(c_{yx},N)=\frac{{T^{\prime }}_{<X>\to <Y>}(c_{yx},N)-{T^{\prime }}_{<X>\to <Y>}(c_{yx},1)}{{T^{\prime }}_{<X>\to <Y>}(c_{yx},1)}$$

We see in Figure 8F that the changes of Granger causality with *c*_*yx*_ are different for different *N*. This means that if we want to compare different connections with different strength (determined by *c*_*yx*_), the results will be affected by the degree of spatial aggregation. However, as illustrated in Figure 8F the influence of changes in the actual coupling strength *c_yx_* is low compared to the influence of the intra-areal integration, as shown in Figure 8E. These results were robust for other configurations of the setup represented in Figure 8D.

Altogether, we have shown that spatial aggregation can produce inconsistent positives of a high relative magnitude, and renders the measures of connectivity particularly sensitive to intra-areal properties, because these properties determine the resulting signals after spatial aggregation.

## Discussion

We started by reviewing previous work about causal inference, comprising Granger causality (Granger, 1980) and causal models (Pearl, 2009). In particular, we described how causal models are associated with graphical causal structures, we indicated that Dynamic Causal Models (DCM) (Friston et al., 2003) are subsumed in the causal models described by Pearl, and that Pearl’s approach does not exclude feedback connections because feedback interactions can be represented in acyclic graphs once the temporal dynamics are explicitly considered. Furthermore, we reviewed the criterion of d-separation to graphically read conditional independencies, and the algorithms proposed by Pearl and collaborators (Pearl, 2009) for causal inference without (IC algorithm) and with (IC^*^ algorithm) the existence of latent variables being considered. These algorithms have as output a graphical pattern that represents the class of all observationally equivalent causal structures compatible with the conditional independencies present in the data.

We then investigated the application of these algorithms to infer causal interactions between dynamic processes. We showed that Granger causality is subsumed by the IC algorithm. From our analysis it is also clear that other recent proposals to decompose Granger causality in different contributions or to identify the delay of the interactions (Runge et al., 2012; Wibral et al., 2013) are also subsumed by the IC algorithm. Moreover, we illustrated that the IC^*^ algorithm provides an output representation not suited for the analysis of dynamic processes, since it assumes the lack of structure of the latent variables. Accordingly, we proposed an alternative new algorithm based on the same principles of the IC^*^ algorithm but specifically designed to study dynamic processes. We did not conceive the new algorithm intending to outperform the IC^*^ algorithm, whose performance is theoretically optimal given the bounds imposed by the existence of observationally equivalent classes. Rather the new algorithm intends to provide a more appropriate and concise representation of the causal structures for dynamic processes. Furthermore, the algorithm integrates Pearl’s algorithmic approach with the use of Granger causality. To our knowledge, this new algorithm is the first to use Granger causality explicitly considering the existence of latent processes. This improvement can be very helpful to assess how informative are the observed Granger causality relations to identify the actual causal structure of the dynamics.

Furthermore, we showed that an adequate graphical model of the setup in which some data are recorded is enough to predict, without any numerical calculation, the existent Granger causality relationships using d-separation. We used this graphical analysis to explain, in a unified way, scenarios in which inconsistent positives of Granger causality have been reported. These comprise non-stationary correlated trends (Lütkepohl, 2005), related ongoing state variability (Wang et al., 2008), discretization (Kaiser and Schreiber, 2002), measurement noise (Nalatore et al., 2007), hemodynamic responses (Deshpande et al., 2010), time aggregation (Granger, 1963; Valdes-Sosa et al., 2011), and spatial aggregation. Regarding the effect of hemodynamic responses, our results may seem contradictory to the recent study of Seth et al. (2013) which shows that Granger causality is invariant when the hemodynamic response is an invertible filter. We note that the graphical analysis with d-separation is suited for stochastic variables, such as the ones in the causal models described in section “Models of Causality.” The invariance of Granger causality is lost if noise variability is incorporated to the hemodynamic response.

We specifically focused on the effect of spatial aggregation of the underlying neural sources between which the causal interactions occur. The effects of spatial aggregation concern virtually all measures of causation calculated from neuroimaging data, and to those obtained with intracranial massed signals such as LFP. Yet, to our knowledge, this problem still remains to be fully understood. We showed that spatial aggregation can induce inconsistent positive Granger causality values of a magnitude comparable to the consistent ones. More generally, it renders Granger causality particularly sensitive to the degree of integration of the processes spatially aggregated. This means that in the presence of spatial aggregation Granger causality, independently of being used for causal inference or as a measure of functional connectivity (Valdes-Sosa et al., 2011; Friston et al., 2013), may reflect more the intra-areal properties of the system than inter-areal interactions.

In this work we followed the framework of Pearl based on causal models and associated graphical causal structures, in which a non-parametric approach to causal inference is proposed that is based on evaluating conditional independencies. In neuroscience applications, and in particular in fMRI analysis, there has been a recent controversy comparing Granger causality and DCM (Valdes-Sosa et al., 2011; Friston et al., 2013). We pointed out that both approaches are theoretically subsumed by Pearl’s framework. In fact, much more relevant than this comparison is the distinction between non-parametric causal inference and model-based causal inference. Granger causality can be calculated in a model-based way, with autoregressive or more refined models (Lütkepohl, 2005), or it can be estimated in a non-parametric way using transfer entropy (e.g., Besserve et al., 2010). The motivation of using a generative model of the observed signals from underlying processes, which is at the core of DCM, is the same of proposing Kalman filters to improve the estimation of Granger causality (Winterhalder et al., 2005; Nalatore et al., 2007).

All the considerations regarding the limitations of causal inference due to observational equivalence and latent variables also hold for model-based approaches like DCM. In DCM the identification of the model causal structure is partially done a priori, by the selection of the priors of the parameters in the model, and partially carried out together with the parameters estimation. Therefore, the model selected (and thus the corresponding causal structure) is not chosen only based on capturing the conditional independencies observed in the data, but also on optimizing some criterion of fitting to the actual data. Given the sophistication of the procedure of model inference, it is not straightforward to evaluate how the selected DCM model reflects the observed conditional independencies (and this may vary across different types of DCM models). Furthermore, the framework of network discovery within DCM (Friston et al., 2011) is very powerful evaluating the posterior probability—evidence- for different models, but still does not incorporate an evaluation of the influence of latent variables, like they do the algorithms of causal inference.

Modeling goes beyond causal inference. A good model gives us information not only about the causal structure, but also about the actual mechanisms that generate the dynamics. But a model can be good in terms of statistical prediction without being an appropriate causal model. That is, the effect of latent processes can be captured indirectly so that the parameters reflect not only the interactions between the observed processes but also the hidden ones. Therefore, even if by definition inside-model causality is well-defined in any DCM model, obtaining a good causal model is much harder than a good statistical model, and cannot be evaluated without interventions on the system. This means that, in the same sense that the Granger causality measures are measures of functional connectivity which, in some cases, can be used to infer causal relations, DCM models are functional connectivity models which, to the extent to which they increasingly reproduce the biophysical mechanisms generating the data, converge to causal models.

The issue of spatial aggregation we addressed here is particularly relevant for causal models, and not only to infer the causal structure. This is because it regards the nature of each node in the graph and requires understanding how causal mechanisms that certainly operate at a finer scale can be captured and are meaningful for macroscopic variables. That is, to which degree can we talk about a *causal* model between variables representing the activity of large brain areas? This is a crucial question for the mechanistical—and not only statistical—interpretation of DCM models, which, despite their increasing level of biological complexity, necessarily stay at a quite macroscopic level of description.

### Conflict of interest statement

The authors declare that the research was conducted in the absence of any commercial or financial relationships that could be construed as a potential conflict of interest.
