# Supplementary material for: Algorithms of causal inference for the analysis of effective connectivity among brain regions
Source: Front Neuroinform. 2014 Jul 2;8:64. doi: 10.3389/fninf.2014.00064 (PMC4078745; doi:10.3389/fninf.2014.00064)
Supplement: Supplementary file 1 [file DataSheet1.PDF]

Supplementary information for "Algorithms of causal inference for the analysis of effective connectivity among brain regions"

The effect of common drivers and mediators on Granger causality and instantaneous causality

In Section "Causal inference with latent variables with dynamic processes" of the Results, we discussed how the existence of a common driver or a mediator in a system composed of processes X and Y is reflected in the Granger causality and instantaneous causality relationships. In particular, Eq. (11) indicates that a mediator induces only Granger causality, while Eq. (12) indicates that a common driver induces both Granger causality and instantaneous causality. This difference generalizes to multivariate systems with any number of observed or latent processes, as we prove here. We provide the generalization and the proof for each of the four conditions presented in Eqs (11, 12). First, for common drivers the sufficient condition involving Granger causality in Eq. (12) generalizes to

$$X \leftarrow \alpha \rightarrow Y \Rightarrow T_{X\rightarrow Y|S} > 0 \;\; \forall S \,, \qquad \text{(S1)}$$

where S is any set of observable processes of the system.

Proof: To prove this sufficient condition we start with the case of Eq. (12), in which the system is composed only by X and Y together with the latent common driver, that is, S is the empty set. The system can be represented in the mesoscopic scale like in Figure 4E, substituting Z by α. One can then examine graphically if Y_{i+1} and X^i are d-separated by $\{Y^i\}$ applying the procedure described in Section "Analysis of the effect of latent variables" and check that they are not d-separated. This is because the path $Y_{i+1} \leftarrow \alpha^i \rightarrow X^i$ cannot be blocked since α is latent. Since the condition of d-separation does not hold, Y_{i+1} and X^i are conditionally dependent given $\{Y^i\}$, which means that $T_{X\rightarrow Y}>0$. Notice however, that the assumption of internal causal influences of the latent process is required to assure that this conditional dependence always holds. In particular, suppose that α is a common driver of X and Y but the lag of the causal connections $\alpha \rightarrow X$ and $\alpha \rightarrow Y$ differs. That is, for example the influences are such that $\alpha_i \rightarrow Y_{i+1}$ and $\alpha_i \rightarrow X_{i+2}$. In this case, the lack of internal causality of α_i would destroy the path $Y_{i+1} \leftarrow \alpha^i \rightarrow X^i$.

We now consider a multivariate system including other observable and latent processes. Accordingly, the conditional transfer entropy can be calculated conditioning on any set S of observable processes. However, even if conditioning on other processes can block other paths related to other common drivers, no conditioning can block the path mentioned above involving the latent α^i, and thus $T_{X\rightarrow Y|S}>0 \; \forall S$. □

Also for a common driver the sufficient condition involving instantaneous causality is

$$X \leftarrow \alpha \rightarrow Y \Rightarrow T_{X\cdot Y|S} > 0 \;\; \forall S \,, \qquad \text{(S2)}$$

where S is any set of observable processes of the system.

Proof: The proof is analogous to the one of Eq. (S1). In the system with only X, Y, and α again by d-separation one can check that the path $Y_{i+1} \leftarrow \alpha^i \rightarrow X_{i+1}$ cannot be blocked, and thus Y_{i+1} and X_{i+1} are conditionally dependent given $\{X^i, Y^i\}$, which means that $T_{X \cdot Y}>0$. Again internal causality of the latent process is required in order to assure that this path always exists. For a multivariate system conditioning on a set of other processes S will never block this path neither. □

For the case of a mediator the sufficient condition of Granger causality generalizes to

$$X \rightarrow \alpha \rightarrow Y \Rightarrow T_{X \rightarrow Y|S} > 0 \ \forall S \ , \qquad \text{(S3)}$$

where S is any set of observable processes of the system.

Proof: Again the proof uses d-separation. In the system with only X, Y, and α the path $X^i \rightarrow \alpha^i \rightarrow Y_{i+1}$ can never be blocked since α is a latent process. In multivariate systems, even if S^i blocks other paths between Y_{i+1} and X^i, this path cannot be blocked. □

The last, instantaneous causality for a mediator, differs from the others. As indicated in Eq. (11), when the system is composed of only X, Y, and α, the mediator leads to $T_{X \cdot Y}=0$. In a multivariate system, in which other processes, observable or latent, can act as common drivers, a zero instantaneous causality can only be recovered finding the proper set S that blocks these other common drivers without activating new ones (in general, notice that conditioning on S^i can block some paths but also activate others if some colliders belong to S^i). Accordingly, the direction of the sufficient condition between the existence of links and dependencies is reversed:

$\exists S \mid T_{X \cdot Y|S}=0 \ \Rightarrow$ There is no latent common driver ($X \leftarrow \alpha \rightarrow Y$) and it is possible that there is a mediator ($X \rightarrow \alpha \rightarrow Y$) (S4)

Proof: Again we start with the system with only X, Y, and α as a mediator. Applying d-separation it can be checked that X^i blocks any path to X_{i+1}, proving the sufficient condition in Eq. (11). For a multivariate set, any mediator will continue without creating instantaneous causality by itself. Two mediators can create instantaneous causality if $X \rightarrow \alpha_1 \rightarrow Y$, $Y \rightarrow \alpha_2 \rightarrow X$, and α_1 and α_2 are correlated, but in this case at least one of them (or a common driver of them) is a common driver of X and Y. Accordingly, instantaneous causality can only arise because of common drivers. The only way to cancel the effect of these common drivers is to condition on them to block the corresponding paths. Since a sufficient condition is equivalent to a sufficient condition in the opposite direction with negative arguments, from Eq. (S2) we have $\exists S \mid T_{X \cdot Y|S}=0 \ \Rightarrow$ There is no latent common driver ($X \leftarrow \alpha \rightarrow Y$), which combined with the fact that mediators do not induce instantaneous causality proves Eq. (S4). □

After proving the generalization of Eqs (11, 12) of Section "Causal inference with latent variables for dynamic processes", we now prove Eq. (13) which we write again here:

$\forall S \ T_{X \cdot Y|S}>0 \Leftrightarrow$ common driver latent processes cause the instantaneous causality, (S5)

where one or more common driver latent processes may be involved.

Proof: This if and only if condition results from Eq. (S4) considering that apart from mediators, direct causal interactions do not create instantaneous causality neither, so that only common drivers induce it. □

The motivation of the ICG* algorithm

As in the IC* algorithm, the stability assumption (see Methods) is used to derive connections from dependencies. Given that, the sufficient condition $\Rightarrow$ of Eq. (S5) justifies Step 1 of the ICG* algorithm. However, it should be noticed that Eq. (S5) does not assure that the latent processes responsible for the instantaneous causality are common drivers of X and Y, although this will most often be the case. In particular, some combination of observable and latent common drivers can lead to $\forall S\ T_{X\cdot Y|S} > 0$ without the existence of a latent process which is a common driver of X and Y. Consider an observable process Z which is a common driver of X and Y ($X \leftarrow Z \rightarrow Y$). At the same time consider two latent processes α_1 and α_2 such that α_1 is a common driver of X and Z ($X \leftarrow \alpha_1 \rightarrow Z$) and α_2 is a common driver of Y and Z ($Z \leftarrow \alpha_2 \rightarrow Y$). In this case without conditioning on Z the common driver Z leads to $T_{X\cdot Y} > 0$, but conditioning on Z activates the path $X \leftarrow \alpha_1 \rightarrow Z \leftarrow \alpha_2 \rightarrow Y$, where Z is a collider. This example is analogous to the one shown in Figure 3E, where Z was also a noncollider and a collider simultaneously depending on the path. Therefore, a bidirectional arrow in the output of the ICG* algorithm can also in some cases result from this combination of common drivers. This indicates that, as it occurs for the IC* algorithm, not all the causal structures in a dependency class share the same skeleton.

Steps 2 and 3 of the ICG* algorithm are justified by Eq. (S5) together with Eq. (S3). Once in Step 1 it is checked that it does not exist a latent common driver, Granger causality can only be produced by a direct causal interaction or by a latent mediator. Analogously to the IC* algorithm these two cases are represented together by a unidirectional arrow. In fact, in contrast to the case in which temporal dynamics are not considered, it is possible in principle to discriminate between a direct influence and an influence mediated by a latent mediator. For example, consider again the system composed only by X and Y with α as a latent mediator of the causal influence from X to Y, against the system with only a direct causal connection from X to Y. In the first case, since causality cannot be instantaneous neither from X to α, nor from α to Y, we have that Y_{i+1} and X^i are conditionally independent given Y^{i-2}. Conversely, if the interaction is direct this conditional independence is only achieved given Y^{i-1}. We here do not exploit this discrimination which we leave for a future refinement of the algorithm, together with the relaxation of the assumption of internal causal connections between the past and future of the latent processes.

Graphical sufficient conditions for Granger causality and instantaneous causality

In Section “Causal inference with latent variables for dynamic processes”, together with the ICG* algorithm, we provided the corresponding graphical sufficient conditions to read the conditional independencies associated with the Granger causality and instantaneous causality relationships from the output graph. The existence of a criterion of separation is what renders the graph a meaningful representation of the conditional independencies. Here we prove the validity of the application of d-separation to the output graphs of the ICG* algorithm, and the validity of the sufficient conditions described in the main text.

As we wrote in the main text, d-separation is applied considering a collider on a path to be any node with two head to head arrows on the path, where the heads can belong to the two types of arrows, i.e, unidirectional or bidirectional.

Graphical sufficient condition for Granger noncausality

X is d-separated from Y by S on each path between X and Y with an arrow pointing to Y $\Rightarrow$ $T_{X\rightarrow Y|S}=0$. (S6)

Graphical sufficient condition for instantaneous noncausality

X is d-separated from Y by S on each path between X and Y with an arrow pointing to X and an arrow pointing to Y $\Rightarrow$ $T_{X\cdot Y|S}=0$. (S7)

Proof: Verma (1993) provides a complete proof of how d-separation can be used to read conditional independencies from DAGs. The output graphs of the ICG* algorithm are not DAGs, since they contain also bidirectional arrows, and are not the causal structure of the system of dynamic processes, since they represent the processes at a macroscopic level. However, as a representation of a dependency equivalent class, each graph is compatible with a set of microscopic graphs which are DAGs and actually are the causal structures of a system of processes. Therefore, to justify the validity of the criterion of separation and the sufficient conditions that we propose for the ICG* output graph, we only need to show that applying them is equivalent to applying d-separation to any of the causal structures of the class they represent.

The conditional independencies associated with Granger causality or instantaneous causality always involve a separation between nodes in the future, at time *i*+1, and sets of nodes in the past. Accordingly, it is enough to consider the mesoscopic level to consider the application of d-separation. To get the mesoscopic graph the following procedure needs to be carried out:

1. Extend the macroscopic graph substituting each bidirectional link by an explicit common driver.
2. Take each node X_k, including the nodes added in 1, and duplicate it to separate a node X_{ki+1} for the future and a node X_k^i for the past.
3. Connect by an arrow the past and the future of all processes, $X_k^i \rightarrow X_{ki+1}$.
4. For any link $X_{k'}\rightarrow X_k$ in the macroscopic graph, add two links $X_{k'}^i\rightarrow X_k^i$ and $X_{k'}^i\rightarrow X_{ki+1}$ in the mesoscopic graph.

Notice that in point 3 internal causality is imposed to all processes, although the assumption of the ICG* algorithm only regards the internal causality of the latent processes. This is not a problem for applying d-separation to check the conditional independencies associated with Granger causality and instantaneous causality. In particular, Granger causality $T_{X_{k'} \rightarrow X_k | S}$ is related to d-separation $X_{ki+1} \perp X_{k'}^{i}\ |X_k^i S^i$, while instantaneous causality $T_{X_{k'} \cdot X_k | S}$ is related to d-separation $X_{ki+1} \perp X_{k'i+1}\ |X_k^i X_{k'}^i S^i$. In both cases each future node which d-separation from another node is examined has the corresponding past node in the blocking set of nodes, so that the links added in point 3 will never be active.

We need to determine when some active path exists so that the separations stated above do not hold. Since the nodes corresponding to the future *i*+1 are not linked directly between them, any path from process *k*' to process *k* goes through other processes through the past nodes and only the last link from *k*'' (where *k*'' can be *k*' itself) is such that $X_{k''}^{i} \rightarrow X_{ki+1}$ exists. Accordingly, considering point 4, an arrow pointing to X_k in the macroscopic graph is required in order for a path to X_{ki+1} to exist. This explains why an arrow pointing to Y is required in (S6) and arrows pointing to X and Y are required in (S7). Furthermore, this last link $X_{k''}^{i} \rightarrow X_{ki+1}$ or any other link between past nodes which belongs to an active path, only exists if and only if a corresponding link exists in the macroscopic graph. This is because they have been added in application of point 4. Therefore, considering that a node $X_{k''}$ blocks or activates a path in the macroscopic graph is equivalent to considering that $X_{k''}^{i}$ blocks or activates a path in the mesoscopic graph. Finally, the extended definition of colliders considers point 1, in which bidirectional arrows are removed by explicitly adding latent common drivers. □

A sufficient condition to infer genuine causation from observations

As we mentioned in Section "Causal inference with latent variables: the IC* algorithm", the Step 3R1 of the IC* algorithm implicitly contains a sufficient condition to infer a genuine causal relation without the necessity to manipulate the system. This sufficient condition of genuine causation can be formulated explicitly as following (Verma, 1993; Pearl, 2009):

Sufficient condition for genuine causation:

A variable X has a genuine causal influence on a variable Y if there is a variable Z and a context S such that:

- *i)* *X and Y are dependent in any context*
- *ii)* *Z is a potential cause of X, i.e., is a genuine cause or they are connected by a common driver.*
- *iii)* *Z and Y are dependent given S.*
- *iv)* *Z and Y are independent given* $S \cup X$.

Notice that the inference of a genuine causal influence involves at least an extra variable apart from X and Y, i.e, Z. As indicated by Pearl (2009) (in definition 2.7.4), the use of temporal precedence simplifies the sufficient condition of Genuine causation:

Sufficient condition for genuine causation (with temporal information)

A variable X has a genuine causal influence on a variable Y if there is a variable Z precedent to X in time and a context S precedent or contemporaneous to X in time such that:

i) *Z and Y are not conditionally independent given S.*
ii) *Z and Y are conditionally independent given* $S \cup X$.

Here we have slightly extended the sufficient condition provided in Pearl (2009) adding the possibility that the variables in S are contemporaneous to X. The possibility of contemporaneous variables is not explicitly mentioned in Verma (1993) or Pearl (2009), but it can be checked that the proof of the sufficient condition provided in Verma (1993) is still valid after this addition. Considering contemporaneous variables in S is relevant for the case of dynamic processes. For example, in Figure 5A genuine causation from Y_i to X_{i+1} is established taking Z = Y_{i-1} and S=X_i, where X_i is contemporaneous to Y_i.

Furthermore, as a especial application of the sufficient condition for genuine causation with temporal information, it is possible to formulate a sufficient condition of genuine causation between processes based on Granger causality:

Sufficient condition for genuine causation between dynamic processes using Granger causality

A process X has a genuine causal influence on a process Y if there is a process Z and a set of processes S such that:

i) *Z is Granger causal to Y given S*
ii) *Z is Granger noncausal to Y given* $S \cup X$.

Proof: The sufficient condition for genuine causation with temporal information should be applied taking X = X^i, Z = Z^i, Y = Y_{i+1}, and S = {Y^i, S^i} □

This sufficient condition for genuine causation using Granger causality illustrates that, when latent variables can exist, a genuine causation can only be inferred testing two Granger causal relationships. To our knowledge, this sufficient condition has not been exploited yet when applying Granger causality to neural data, in the same way that latent processes have not been considered explicitly, in contrast to what we propose in the ICG* algorithm.

The implementation of the algorithms of causal inference to analyze experimental data

A general assumption in the work of Pearl on causal inference is that the joint probability of all the variables in the graph is perfectly estimated. This of course is not generally the case

when analyzing experimental data, in particular when dealing with time series for which the set of variables involved in the analysis comprises the sampling of the past of the processes. Accordingly, an implementation of the IC and IC* algorithms requires complementing the algorithms with a concrete way to evaluate the conditional independencies examined in Step 1. The same occurs for the here proposed ICG* algorithm, in which conditional independencies are assessed calculating Granger and instantaneous causalities in Steps 1-3.

In this article we have not focused on any particular implementation of the algorithms. We mostly evaluated the conditional independencies directly from the causal structures, using the sufficient condition that implies conditional independence from d-separation. When we calculated Granger causality measures (Section "The case of spatial aggregation") we did the calculation analytically from the covariance matrix.

In this Section we discuss the implementation steps necessary to apply the algorithms to experimental data. Even if a complete implementation requires addressing the points mentioned below, we should notice that these steps are not specific for the algorithms of causal inference, but rather represent general issues with hypothesis testing, in particular tests of conditional independencies, on real data. We deliberately separated the exposition of the principles of the existing algorithms and of the new algorithm from the practical implementation problems to facilitate the appreciation of the specific characteristics of causal inference, of the fundamental limitations regarding the consideration of latent variables, and of the comparison with methodologies commonly used to study effective connectivity such as Granger causality and Dynamic Causal Modeling. Moreover, we here do not intend to determine the optimal way to implement the algorithms, but to discuss which elements of this implementation are common or different for the algorithms and the direct application of Granger causality.

The steps of implementation can be classified in a few categories, as following:

Selection of a measure to test for conditional independence

Conditional independencies can be tested in general using conditional mutual information. However, other statistics may be preferable depending on the data set because they are easier to estimate, even if they only test particular differences in the probability distributions compared. For example, the Tetrad program (Spirtes et al., 2000) implements algorithms analogous to the IC* algorithm using second order statistics to test conditional independencies, so that the linear coefficient of partial correlation is used instead of conditional mutual information. Similarly, Granger (Granger, 1969) used a linear implementation comparing prediction errors in order to test Granger causality, so that only the mean of the distributions in Eq. (5) is compared, and not the whole distributions.

Estimation of the measures and assessment of significance

The details of the estimation vary for different measures, and the same measure can be estimated in different ways. For example, the linear Granger causality measures can be estimated non-parametrically or modeling the signals as an autoregressive process. Similarly, there is a vast literature on the practical estimation of mutual information (e.g. Panzeri et al., 2007) and transfer entropy (e.g. Hlaváčková-Schindler et al., 2007). These

measures require the sampling of high-dimensional probability distributions which produces some estimation bias. Accordingly, the estimation of the measures has to be complemented with a procedure to assess if the value obtained is considered significantly different from zero, thus indicating the existence of conditional dependence. For some linear measures it is possible to obtain analytical levels of significance, (e.g. Ramb et al. 2013 and references therein); however, in general surrogate data are needed to provide these levels (e.g. Roebroeck et al., 2005; Besserve et al., 2010). These issues are common to both algorithms and to Granger causality, since they generally concern testing hypotheses of conditional independence. An advantage of the ICG* algorithm is that it allows to examine the causal interactions at the macroscopic level, thus reducing the number of conditional independencies which significance needs to be assessed.

Optimization of the set S_{ab} of conditioning variables

The algorithms as well as the direct application of Granger causality require the selection of conditioning variables. Even in the simplest case of bivariate Granger causality, one should in principle condition on the whole past of the processes, which is practically unfeasible. For the transfer entropy it has been common to condition just in one time lag of the past (Schreiber, 2000). However, different strategies have been proposed to select which time lags are included (e.g. Faes et al., 2011), or to compensate the limited sampling of the past using general variables reflecting the state of the system (Stetter et al. 2012).

For multivariate settings in which conditional mutual information and conditional Granger causality measures are required the selection of the conditioning variables also implies choosing which third processes are used for conditioning. In the direct application of conditional Granger causality it is common to consider that the conditioning should be done on the whole rest of the processes in the system, which is unfeasible in terms of estimation when large networks are studied. Strategies for the selection of the conditioning processes have been proposed (e.g. Marinazzo et al., 2012). For the algorithms of causal inference, which in principle may need to screen all possible sets S_{ab} of all sizes, the common procedure is to start from the smallest size and increase it until a zero value is obtained. Possible strategies to select among the sets of equal size are common to the ones proposed for Granger causality (Faes et al., 2011; Marinazzo et al., 2012).

Given this sketch of the steps required in order to implement the algorithms of causal inference it is clear that the validity of any output graph from the algorithms depends on the proper estimation of the measures and on the significance assessment. Therefore, different significance levels can generally lead to changes in the output causal pattern. The particular way to take this into account for the validation and interpretation of the results is left for a future contribution.

References

Besserve, M., Scholkopf, B., Logothetis, N.K., and Panzeri, S. (2010). Causal relationships between frequency bands of extracellular signals in visual cortex revealed by an information theoretic analysis. *Journal of Computational Neuroscience* 29**,** 547-566. doi: 10.1007/s10827-010-0236-5

Faes, L., Nollo, G., and Porta, A. (2011). Information-based detection of nonlinear Granger causality in multivariate processes via a nonuniform embedding technique. *Physical Review E* 83:051112. doi: 10.1103/PhysRevE.83.051112

Granger, C. W. J. (1969). Investigating causal relations by econometric models and cross-spectral methods. *Econometrica* 37(3), 424-438. doi: 10.2307/1912791

Hlaváčková-Schindler, K., Paluš, M., Vejmelka, M., Bhattacharya, J. (2007). Causality detection based on information-theoretic approaches in time series analysis. *Physics Reports* 441(1), 1-46. doi: 10.1016/j.physrep.2006.12.004

Marinazzo, D., Pellicoro, M., and Stramaglia, S. (2012). Causal Information Approach to Partial Conditioning in Multivariate Data Sets. *Computational and Mathematical Methods in Medicine* 303601. doi: 10.1155/2012/303601

Panzeri, S., Senatore, R., Montemurro M. A., and Petersen, R. S. (2007). Correcting for the sampling bias problem in spike train information measures. *J Neurophysiol* 98, 1064-1072. doi: 10.1152/jn.00559.2007

Pearl, J. (2009). *Causality: Models, Reasoning, Inference.* New York: Cambridge University Press.

Ramb, R., Eichler, M., Ing, A., Thiel, M., Weiller, C., Grebogi, C., Schwarzbauer, C., Timmer, J., and Schelter, B. (2013). The impact of latent confounders in directed network analysis in neuroscience. *Philosophical Transaction of the Royal Society A* 371:20110612. doi:10.1098/rsta.2011.0612].

Roebroeck, A., Formisano, E., and Goebel, R. (2005). Mapping directed influence over the brain using Granger causality and fMRI. *Neuroimage* 25**,** 230-242. doi: doi:10.1016/j.neuroimage.2004.11.017

Schreiber, T. (2000). Measuring information transfer. *Physical Review Letters* 85**,** 461-464. doi: 10.1103/PhysRevLett.85.461

Spirtes, P., Glymour, C.N., and Scheines, R. (2000). *Causation, Prediction, and Search.* Cambridge, MA: MIT Press.

Verma, T. (1993). Graphical aspects of causal models, Technical Report R-191, UCLA, Computer Science Department.
